# Genome-Wide Association Study in East Asians Identifies Novel Susceptibility Loci for Breast Cancer

**DOI:** 10.1371/journal.pgen.1002532

**Published:** 2012-02-23

**Authors:** Jirong Long, Qiuyin Cai, Hyuna Sung, Jiajun Shi, Ben Zhang, Ji-Yeob Choi, Wanqing Wen, Ryan J. Delahanty, Wei Lu, Yu-Tang Gao, Hongbing Shen, Sue K. Park, Kexin Chen, Chen-Yang Shen, Zefang Ren, Christopher A. Haiman, Keitaro Matsuo, Mi Kyung Kim, Ui Soon Khoo, Motoki Iwasaki, Ying Zheng, Yong-Bing Xiang, Kai Gu, Nathaniel Rothman, Wenjing Wang, Zhibin Hu, Yao Liu, Keun-Young Yoo, Dong-Young Noh, Bok-Ghee Han, Min Hyuk Lee, Hong Zheng, Lina Zhang, Pei-Ei Wu, Ya-Lan Shieh, Sum Yin Chan, Shenming Wang, Xiaoming Xie, Sung-Won Kim, Brian E. Henderson, Loic Le Marchand, Hidemi Ito, Yoshio Kasuga, Sei-Hyun Ahn, Han Sung Kang, Kelvin Y. K. Chan, Hiroji Iwata, Shoichiro Tsugane, Chun Li, Xiao-Ou Shu, Dae-Hee Kang, Wei Zheng

**Affiliations:** 1 Division of Epidemiology, Department of Medicine, Vanderbilt Epidemiology Center, Vanderbilt-Ingram Cancer Center, Vanderbilt University School of Medicine, Nashville, Tennessee, United States of America; 2 Department of Biomedical Sciences, Seoul National University College of Medicine, Seoul, Korea; 3 Shanghai Center for Disease Control and Prevention, Shanghai, China; 4 Department of Epidemiology, Shanghai Cancer Institute, Shanghai, China; 5 Department of Epidemiology and Biostatistics, Nanjing Medical University, Nanjing, China; 6 Department of Preventive Medicine, Seoul National University College of Medicine, Seoul, Korea; 7 Cancer Research Institute, Seoul National University College of Medicine, Seoul, Korea; 8 Department of Epidemiology and Biostatistics, Tianjin Medical University Cancer Institute and Hospital, Tianjin, China; 9 Institute of Biomedical Sciences, Academia Sinica, Taipei, Taiwan; 10 College of Public Health, China Medical University, Taichung, Taiwan; 11 School of Public Health, Sun Yat-sen University, Guangzhou, China; 12 Department of Preventive Medicine, Keck School of Medicine, University of Southern California and Norris Comprehensive Cancer Center, Los Angeles, California, United States of America; 13 Division of Epidemiology and Prevention, Aichi Cancer Center Research Institute, Nagoya, Japan; 14 Cancer Epidemiology Division, Breast Cancer Center, National Cancer Center, Goyang, Korea; 15 Department of Surgery, Asan Medical Center, University of Ulsan College of Medicine, Ulsan, Korea; 16 Department of Pathology, Li Ka Shing Faculty of Medicine, University of Hong Kong, Hong Kong, China; 17 Epidemiology and Prevention Division, Research Center for Cancer Prevention and Screening, National Cancer Center, Tokyo, Japan; 18 Division of Cancer Epidemiology and Genetics, National Cancer Institute, National Institutes of Health, Department of Health and Human Services, Bethesda, Maryland, United States of America; 19 Department of Surgery, Seoul National University College of Medicine, Seoul, Korea; 20 Center for Genome Science, Korea National Institute of Health, Seoul, Korea; 21 Department of Surgery, Soonchunhyang University Hospital, Bucheon, Korea; 22 Department of Clinical Oncology, Queen Mary Hospital, Hong Kong, China; 23 First Affiliated Hospital, Sun Yat-sen University, Guangzhou, China; 24 Cancer Center, Sun Yat-sen University, Guangzhou, China; 25 Epidemiology Program, Cancer Research Center, University of Hawaii, Honolulu, Hawaii, United States of America; 26 Department of Surgery, Nagano Matsushiro General Hospital, Nagano, Japan; 27 Department of Obstetrics and Gynecology, Li Ka Shing Faculty of Medicine, University of Hong Kong, Hong Kong, China; 28 Department of Breast Oncology, Aichi Cancer Center Central Hospital, Nagoya, Japan; 29 Department of Biostatistics, Vanderbilt University School of Medicine, Nashville, Tennessee, United States of America; University of Oxford, United Kingdom

## Abstract

Genetic factors play an important role in the etiology of both sporadic and familial breast cancer. We aimed to discover novel genetic susceptibility loci for breast cancer. We conducted a four-stage genome-wide association study (GWAS) in 19,091 cases and 20,606 controls of East-Asian descent including Chinese, Korean, and Japanese women. After analyzing 690,947 SNPs in 2,918 cases and 2,324 controls, we evaluated 5,365 SNPs for replication in 3,972 cases and 3,852 controls. Ninety-four SNPs were further evaluated in 5,203 cases and 5,138 controls, and finally the top 22 SNPs were investigated in up to 17,423 additional subjects (7,489 cases and 9,934 controls). SNP rs9485372, near the TGF-β activated kinase (*TAB2*) gene in chromosome 6q25.1, showed a consistent association with breast cancer risk across all four stages, with a *P*-value of 3.8×10^−12^ in the combined analysis of all samples. Adjusted odds ratios (95% confidence intervals) were 0.89 (0.85–0.94) and 0.80 (0.75–0.86) for the A/G and A/A genotypes, respectively, compared with the genotype G/G. SNP rs9383951 (*P* = 1.9×10^−6^ from the combined analysis of all samples), located in intron 5 of the *ESR1* gene, and SNP rs7107217 (*P* = 4.6×10^−7^), located at 11q24.3, also showed a consistent association in each of the four stages. This study provides strong evidence for a novel breast cancer susceptibility locus represented by rs9485372, near the *TAB2* gene (6q25.1), and identifies two possible susceptibility loci located in the *ESR1* gene and 11q24.3, respectively.

## Author Summary

Breast cancer is one of the most common malignancies among women worldwide. Genetic factors play an important role in the etiology of breast cancer. To identify common genetic susceptibility alleles for breast cancer, we performed a four-stage genome-wide association study in 19,091 cases and 20,606 controls among East-Asian women. Single nucleotide polymorphism (SNP) rs9485372, near the TGF-beta activated kinase 1 (*TAB2*) gene at chromosome 6q25.1, was associated with breast cancer risk (*P* = 3.8×10^−12^). SNPs rs9383951, located in intron 5 of the estrogen receptor 1 (*ESR1*) gene, and rs7107217, located at 11q24.3, were also consistently associated with breast cancer risk in all four stages with a combined *P* of 1.9×10^−6^ and 4.6×10^−7^, respectively. This study provides strong evidence for a novel breast cancer susceptibility locus represented by rs9485372, near the *TAB2* gene (6q25.1), and identifies two possible susceptibility loci located in the *ESR1* gene and 11q24.3, respectively.

## Introduction

Breast cancer is one of the most common malignancies diagnosed among women worldwide, including those living in East Asian countries. Genetic factors play an important role in the etiology of both sporadic and familial breast cancer [1]. In the past two decades, more than 1,000 reports have been published addressing the association between variants in candidate genes and breast cancer risk. However, only a few genetic risk factors have been confirmed for this common malignancy [2]. Recent genome-wide association studies (GWAS) have identified approximately 20 common genetic susceptibility loci for breast cancer [3]–[14]. However, these newly-identified genetic factors, along with known high-penetrance breast cancer susceptibility genes explain less than 30% of the heritability for this cancer [2], [15]. Furthermore, most GWAS were conducted among women of European ancestry, and many of the variants discovered in European-ancestry populations showed only a weak or no association with breast cancer in other ethnic groups [16], [17]. For example, only 8 of 12 breast cancer risk SNPs identified in women of European ancestry were directly replicated in Chinese population [18]. Therefore, GWAS conducted in non-European women are needed to fully uncover the genetic basis for breast cancer susceptibility. Herein, we report results from a large GWAS of breast cancer conducted in East Asian women.

## Results

**Figure 1:**
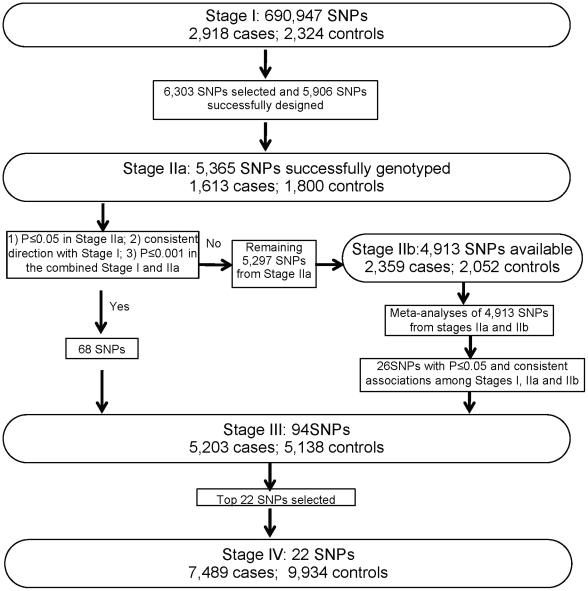
Overview of the study design.

A total of 19,091 female breast cancer cases and 20,606 female controls—including 23,891 Chinese, 11,907 Korean and 3,809 Japanese women—were included in the present study (Table 1). In Stage I, we analyzed 690,947 SNPs in 2,918 breast cancer cases and 2,324 community controls recruited from studies conducted in Shanghai, China (Figure 1, Text S1). Top 5,365 SNPs were investigated in Stage IIa including 1,613 Chinese cases and 1,800 Chinese controls recruited from studies conducted in Shanghai, China. Of the SNPs evaluated, 68 SNPs showed an association with breast cancer risk at *P*≤0.05 with the same direction as observed in Stage I. We performed a meta-analysis for the remaining 4,913 SNPs with data available from both Stage IIa and Stage IIb (2,359 Korean cases and 2,052 Korean controls). Twenty-six SNPs showed an association with breast cancer risk with P_meta_≤0.05 and the association was consistent among Stages I, IIa and IIb. These SNPs, along with the 68 SNPs mentioned above, were selected for Stage III replication in 4,712 cases and 4,496 controls. Finally, based on the results of the first three stages, 22 top SNPs were selected for Stage IV evaluation in 7,489 cases and 9,934 controls.

**Table 1:** Selected characteristics of studies participating in the Asia Breast Cancer Consortium.

Study Stage^a^	Ethnicity	No. of cases	No. of controls	age^b^	Menopause (%)^c^	ER+ (%)
**Stage I**						
Shanghai-I	Chinese	2,918	2,324	51.7/50.3^d^	42.9/41.7	65.3
**Stage II**						
Shanghai-II (IIa)	Chinese	1,613	1,800	53.2/53.4	50.2/55.1	62.5
SeBCS-I (IIb)	Korean	2,359	2,052	48.1/51.7	37.9/52.0	61.9
**Stage III**						
Shanghai-III	Chinese	2,601	2,386	53.8/55.1^d^	50.3/52.6	64.9
Taiwan	Chinese	1,066	1,065	51.5/47.5^d^	52.3/39.9	66.1
Nagoya	Japanese	644	644	51.4/51.1	48.5/48.5	72.8
Nagano	Japanese	401	401	53.8/54.0	54.9/65.3	74.6
**Stage IV**						
Nanjing	Chinese	1,786	1,837	50.6/50.2	51.3/47.6	55.7
Tianjin	Chinese	1,297	1,585	51.9/51.9	51.9/55.5	44.2
Guangzhou	Chinese	838	865	49.0/49.2	41.8/51.9	71.6
NCC	Korean	505	504	49.0/49.1	49.5/45.3	65.0
SeBCS-II	Korean	777	1,104	47.5/47.7	36.3/37.3	63.0
KOHBRA/KoGES	Korean	1,397	3,209	40.5/50.3^d^	23.3/	62.8
MEC	Japanese	889	830	66.5/66.5		85.3
**Total**		19,091	20,606			

a See the methods section for the full names of participating studies.

b Mean value for cases/controls.

c Percentage for cases/controls.

d Significant at α = 0.01 level.

**Figure 2:**
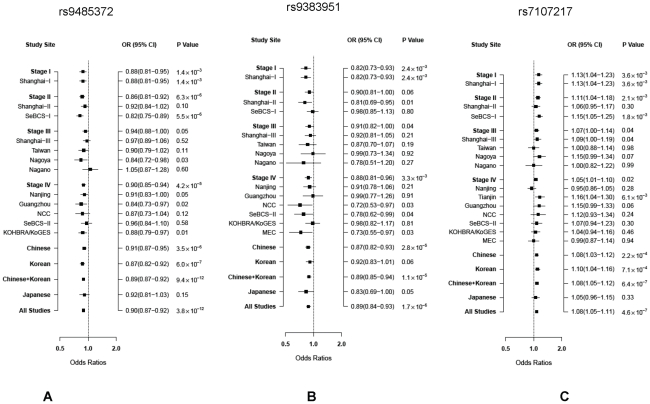
ORs per risk allele and 95% CIs for breast cancer associated with three SNPs by study site and ethnicity. A: rs9485372, B: rs9383951; and C: rs7107217.

SNP rs9485372 showed a statistically significant association with breast cancer risk in each of the four stages (Table 2). The OR (95% CI) per A allele was 0.88 (0.81–0.95), 0.86 (0.81–0.92), 0.94 (0.88–1.00) and 0.90 (0.85–0.94), respectively, for stages I to IV. The association with this SNP was remarkably consistent across all but one small study (Figure 2A). Pooled analysis of samples from all studies produced OR (95% CI) of 0.90 (0.87–0.92) and *P*-value of 3.8×10^−12^, which is substantially lower than the conventional genome-wide significance level of 5×10^−8^ based on conservative Bonferroni adjustment of multiple comparisons at α = 0.05, providing strong evidence for an association of this SNP with breast cancer risk.

**Table 2:** Summary of results for the three SNPs showing a statistically or marginally significant association in all four stages with breast cancer risk, the Asia Breast Cancer Consortium.

SNP^a^	Position^b^	Study	No. of Cases/Controls	EAF (%)^c^	Per allele OR (95%CI) d	P value^d^
rs9485372 (A/G)	149650567 (6q25.1)					
		Stage I	2,770/2,175	43.5	0.88(0.81–0.95)	1.4×10^−3^
		Stage II	3,930/3,818	47.1	0.86(0.81–0.92)	6.3×10^−6^
		Stage III	4,081/4,074	43.2	0.94(0.88–1.00)	0.05
		Stage IV	5,186/7,440	46.2	0.90(0.85–0.94)	4.2×10^−5^
		All stages	15,967/17,507	45.4	0.90(0.87–0.92)	3.8×10^−12^
rs9383951 (C/G)	152337306 (6q25.1)					
		Stage I	2,916/2,319	11.4	0.82(0.73–0.93)	2.4×10^−3^
		Stage II	3,948/3,836	10.1	0.90(0.81–1.00)	0.06
		Stage III	4,581/4,433	9.7	0.91(0.82–1.00)	0.06
		Stage IV	6,117/8,296	9.6	0.88(0.81–0.96)	3.3×10^−3^
		All stages	17,562/18,884	10.0	0.88(0.84–0.93)	1.9×10^−6^
rs7107217 (C/A)	128978900 (11q24.3)					
		Stage I	2,916/2,319	31.4	1.13(1.04–1.23)	3.6×10^−3^
		Stage II	3,929/3,839	34.8	1.11(1.04–1.18)	2.1×10^−3^
		Stage III	4,606/4,424	35.2	1.07(1.00–1.14)	0.04
		Stage IV	7,348/9,831	37.4	1.05(1.01–1.10)	0.02
		All stages	18,799/20,413	35.8	1.08(1.05–1.11)	4.6×10^−7^

a Effect/reference alleles based on forward strand.

b From NCBI genome build 36.

c Effect allele frequency in controls.

d Adjusted for age and study sites.

Two other SNPs, rs9383951 and rs7107217, were also consistently replicated in each of the three replication sets. The C allele of rs9383951 was associated with decreased risk with OR (95% CI) of 0.82 (0.73–0.93), 0.90 (0.81–1.00), 0.91 (0.82–1.00), and 0.88 (0.81–0.96), respectively, for stages I to IV (Table 2). The P-value reached 1.9×10^−6^ in the pooled analysis of samples from all four stages. For SNP rs7107217, the ORs (95% CI) per C allele were 1.13 (1.04–1.23), 1.11 (1.04–1.18), 1.07 (1.00–1.14) and 1.05 (1.01–1.10), respectively, for stages I to IV, respectively (Table 2). Analyses with all subjects combined showed OR (95% CI) of 1.08 (1.05–1.11) and *P* value of 4.6×10^−7^. Again, the association of breast cancer risk with these two SNPs was very consistent across the vast majority of participating studies (Figure 2B and 2C).

Stratified analyses showed that the associations with these three SNPs were consistent in all three East Asian populations, although the association for SNPs rs9485372 and rs7107217 was not significant for Japanese subjects, probably due to a small sample size (Table 3). Associations of these three SNPs with breast cancer risk were similar when stratified by menopausal or estrogen receptor status and none of the heterogeneity tests was statistically significant (Table S1). No significant interaction was observed with other risk factors (Table S1). After adjusted for the top 5 or 10 principal components, the results did not change significantly (Table S2).

**Table 3:** Association of SNPs with breast cancer risk by ethnic groups, the Asia Breast Cancer Consortium.

SNP	Study	No. of Cases/Controls	EAF (%)^a^	OR (95% CI)^b^		P value^b^
				Heterozygote	Homozygote	
rs9485372						
	Chinese	9,922/9,644	43.2	0.90(0.84–0.96)	0.83(0.76–0.90)	3.5×10^−6^
	Korean	5,006/6,825	48.2	0.87(0.79–0.95)	0.76(0.68–0.85)	6.0×10^−7^
	Chinese+Korean	14,928/16,469	45.2	0.89(0.85–0.94)	0.80(0.75–0.85)	9.4×10^−12^
	Japanese	1,039/1,038	47.5	0.93(0.76–1.13)	0.84(0.66–1.07)	0.15
	All studies	15,967/17,507	45.4	0.89(0.85–0.94)	0.80(0.75–0.86)	3.8×10^−12^
rs9383951						
	Chinese	10,625/10,180	10.7	0.86(0.80–0.92)	0.87(0.67–1.13)	3.4×10^−5^
	Korean	5,011/6,833	9.7	0.92(0.83–1.02)	0.79(0.52–1.19)	0.06
	Chinese+Korean	15,636/17,013	10.3	0.88(0.83–0.93)	0.86(0.69–1.07)	1.3×10^−5^
	Japanese	1,926/1,871	6.8	0.86(0.71–1.05)	0.40(0.14–1.13)	0.05
	All studies	17,562/18,884	10.0	0.88(0.83–0.93)	0.83(0.67–1.03)	1.9×10^−6^
rs7107217						
	Chinese	11,887/11,719	32.3	1.09(1.03–1.15)	1.14(1.05–1.25)	2.2×10^−4^
	Korean	4,987/6,824	38.7	1.13(1.04–1.23)	1.19(1.06–1.34)	7.1×10^−4^
	Chinese+Korean	16,874/18,543	34.6	1.10(1.05–1.15)	1.16(1.08–1.24)	6.4×10^−7^
	Japanese	1,925/1,870	47.3	1.09(0.94–1.27)	1.09(0.91–1.31)	0.33
	All studies	18,799/20,413	35.8	1.10(1.05–1.15)	1.15(1.08–1.22)	4.6×10^−7^

a Effect allele frequency in controls.

b Adjusted for age and study sites.

Both SNPs rs9485372 and rs9383951 are located at chromosome 6q25.1, approximately 2.34 Mb and 350 kb from the SNP rs2046210 that we previously reported for breast cancer risk [8]. None of these three SNPs, however, are in LD (r^2^<0.1) in any of the three populations (Asian, European and Africans) as determined using data generated in the HapMap or any of the study populations included in the current study (Table S3 and Figure S1). In an analysis including all 30,153 subjects who were genotyped for three SNPs in 6q25.1, all three SNPs remained strongly associated with breast cancer risk after mutual adjustment of the other 2 SNPs with P values of 1.4×10^−12^, 1.3×10^−4^, and 6.0×10^−39^ for SNPs rs9485372, rs9383951 and rs2046210, respectively (Table S4). No significant interaction was observed for these three SNPs (Table S5). We also created a genetic risk score (GRS) to evaluate the combined effect of three SNPs located in 6q25.1 (Table S6). Compared with women carrying 0–1 risk variants, women carrying 6 variants had over two-fold increased risk with an OR (95% CI) of 2.36 (1.89–2.96) and a P value of 1.3×10^−47^.

A total of 376 SNPs were successfully imputed in the LD blocks including rs2046210 and rs9485372 and the whole *ESR1* gene with RSQ≥0.3 and minor allele frequency (MAF)≥0.05. Among them, 27 SNPs showed an association with breast cancer risk with P≤0.05 after adjusted for age, rs9485372, rs9383951 and rs2046210 (Table S7). With the exception of rs4591859 and rs7776340 in the locus of rs2046210 and rs7768330 in the locus of rs9383921, all other SNPs are in the same LD block within the *ESR1* gene (Figure S2). No additional SNP in the rs9485372 locus showed an association with breast cancer risk at p<0.05 after adjusted for rs9485372, rs2046210, and rs9383921.

## Discussion

In this large GWAS conducted in East-Asian women including 19,091 cases and 20,606 controls, we provided strong evidence for a novel breast cancer susceptibility locus represented by rs9485372 and suggestive evidence for two other loci, represented by SNPs rs9383951 and rs7107217.

**Figure 3:**
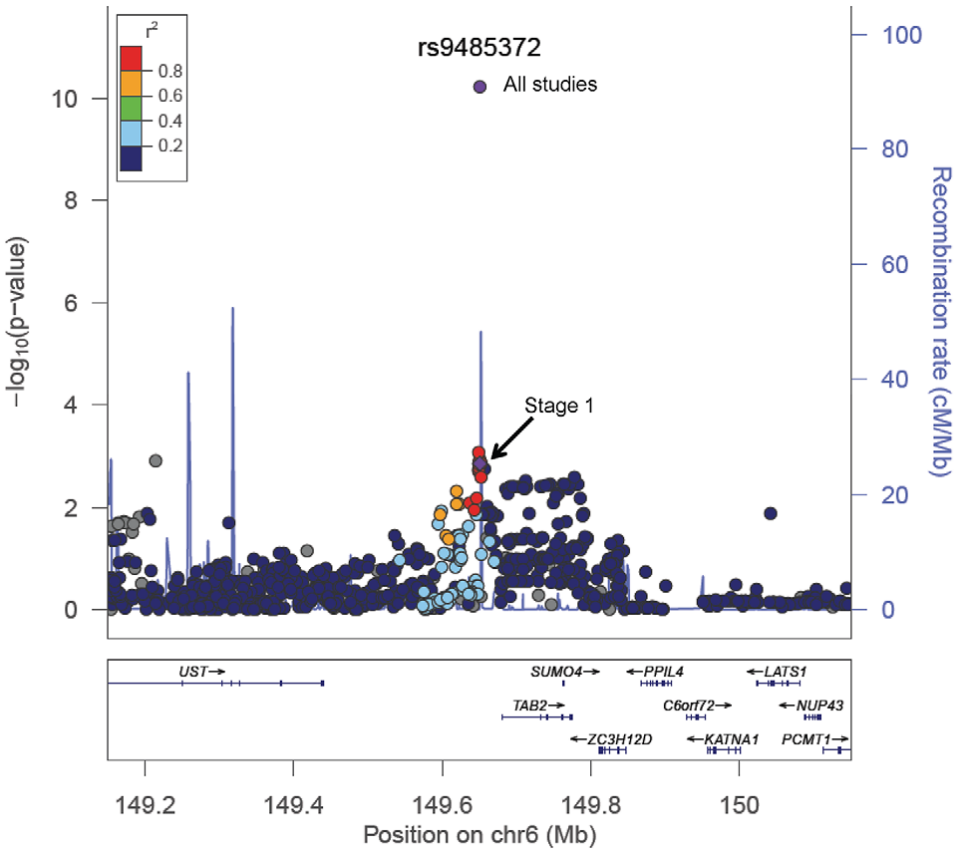
A regional plot of the −log_10_P-values for SNPs at 6q25.1. The LD is estimated using data from HapMap Asian population. Also shown are the SNP Build 36 coordinates in kilobases (Kb), recombination rates in centimorgans (cM) per megabase (Mb) and genes in the region (below) based on the March 2006 UCSC genome browser assembly.

We previously reported a genetic susceptibility locus at 6q25.1, represented by rs2046210, for breast cancer risk [8]. The newly identified SNPs, rs9485372 and rs9383951, also are located at chromosome 6q25.1. However, these three SNPs are not in LD and are thus representing independent breast cancer susceptibility loci. All of them were associated with breast cancer risk after mutual adjustment of the other two SNPs. SNP rs9485372 is approximately 31 Kb upstream of the TGF-β activated kinase 1/MAP3K7 binding protein 2 (*TAB2*) gene (Figure 3). The protein encoded by this gene is an activator of MAP3K7/TAK1, which is required for the IL-1 induced activation of NF-κB and MAPK8/JNK. The TGF-β pathway plays a major role in breast cancer development and progression [19]. The MAP kinases pathway is critical in regulating cell growth and cell death [20] and may contribute to the development of cancer [20]. Furthermore, the TAB2 protein is required for DNA damage-induced TAK1 activation, suggesting that TAB2 may play a role in DNA damage repair [21]. Other genes in the region identified in the study included *SUMO4*, *LATS1*, *PPIL4*, and *UST*. However, given the proximity of the *TAB2* gene with rs9485372 and the important role of this gene in breast carcinogenesis, it is possible that the association between rs9485372 and breast cancer risk may be mediated through the *TAB2* gene. It is also possible that the association may be mediated through regulating the *ESR1* gene, located approximately 2.5 Mb from rs9485372. This possibility was highlighted by a recent study showing that several open reading frames in the 6q25.1 regions co-expressed with *ESR1* [22]. Further research is warranted to clarify the mechanism of the association identified in the study.

SNP rs9383951 is located in intron 5 of the *ESR1* gene, an important gene that has been documented to play a key role in breast cancer development and progression. Previous candidate gene studies have extensively evaluated two SNPs, rs2234693 (Pvull) and rs9340799 (XbaI), in the *ESR1* gene in relation to breast cancer risk; the results, however, have been inconsistent [2]. Neither rs2234693 nor rs9340799 are in LD (r^2^<0.01) with the SNPs discovered in the present study. To follow-up the lead from our previous study reporting a susceptibility locus at 6q25.1 for breast cancer [8], two recent studies conducted among women of European descent identified rs3757318 and rs9397435 in relation to breast cancer risk [11], [23]. These two SNPs are in strong LD (r^2^>0.6 in Asians) with the SNP (rs2046210) we previously reported at 6q25.1 in East Asians but not in other populations. Again, these two SNPs are not in LD (r^2^<0.01 in Asian, European and African populations) with rs9383951 and rs9485372 identified in this study. Although the association with rs9383951 did not reach the conventional genome-wide significance, the fact that this SNP is located in the *ESR1* gene strongly suggests a true association of this SNP with breast cancer risk.

SNP rs7107217 also showed a consistent association in all four stages, although the pooled *P*-value did not reach the conventional genome-wide significance level. This SNP is located at 11q24.3, 152 Kb downstream of the *BARX2* gene and 212 Kb upstream of the *TMEM45B* gene (Figure S3). *BARX2* is a homeobox gene for which the mouse ortholog has been shown to influence cellular processes that control cell adhesion and cytoskeleton remodeling. It has been shown, BARX2 and estrogen receptor-alpha (ESR1) coordinately regulate the production of alternatively spliced ESR1 isoforms and control breast cancer cell growth and invasion [24]. BARX2 also acts in a tumor suppressor and loss of heterozygosity of this gene, lead to poorer survival in patients with ovarian cancer [25].

It could be ideal to increase the sample size in the discovery stage and simplify the replication stages of the study. However, like many other consortium projects, financial constraints and some logistical issues prevented us for achieving the maximum statistical power. Nevertheless, with approximately 40,000 cases and controls, our study represents the largest breast cancer genetic association study in East Asian women. This consortium will continue to provide valuable resources to identify additional novel susceptibility loci for breast cancer.

In summary, in this large GWAS conducted in East Asia women, we provided convincing evidence for an association with a novel independent susceptibility locus located at 6q25.1, near the *TAB2* gene. Our study also suggests that genetic variants in the *ESR1* gene and chromosome 11q24.3 may be related to breast cancer risk. Given that multiple independent breast cancer susceptibility loci have identified in our studies and studies conducted by others in 6q25.1 that harbors the *ESR1* gene, it is possible that 6q25.1 may represent an important region for breast cancer susceptibility.

## Methods

### Study populations

Included in this consortium project were 19,091 cases and 20,606 controls from 14 studies (Table 1). Detailed descriptions of these participating studies and demographic characteristics of study participants are provided in Text S1. Briefly, the consortium included 23,981 Chinese women, 11,907 Korean women, 3,809 Japanese women. The Chinese women were from 8 studies: Shanghai [n = 13,642, Shanghai Breast Cancer Study, Shanghai Breast Cancer Survival Study (SBCSS), Shanghai Endometrial Cancer Study (SECS), Shanghai Women Health Study (SWHS)] [8], , Nanjing (n = 3,623) [27], Tianjin (n = 2,882) [28], Taiwan (n = 2,131) [29], and Guangzhou (n = 1,703). The Korean women were from four studies [Seoul Breast Cancer Study (SeBCS) (n = 6,292) [30], Korea NCC (n = 1,009), KoGES (n = 3,209) [31], and KOHBRA (n = 1,397) [32]]. The Japanese women were from three studies conducted in Hawaii and Los Angeles [n = 1,719; Multiethnic Cohort Study (MEC) [33]], Nagoya (n = 1,288) [34], and Nagano (n = 802) [35] (Table 1). Approval was granted from relevant institutional review boards in all study sites; all included subjects gave informed consent.

### Genotyping methods

The Genotyping protocol for Stage I has been described previously [8]. Briefly, the initial 300 subjects were genotyped using the Affymetrix GeneChip Mapping 500K Array Set. The remaining 4,985 subjects were genotyped using the Affymetrix Genome-Wide Human SNP Array 6.0. We included one negative control and at least three positive quality control (QC) samples from the Coriell Cell Repositories (http://ccr.coriell.org/) in each of the 96-well plates for Affymetrix SNP Array 6.0 genotyping. A total of 273 positive QC samples were successfully genotyped, and the average concordance rate was 99.9% with a median value of 100%. The sex of all study samples was confirmed to be female. Genetically identical, unexpected duplicated samples were excluded, as were close relatives with a pair-wise proportion of identify-by-descent (IBD) estimate greater than 0.25. All samples with a call rate<95% were excluded. The SNPs were excluded if: (i) MAF<1%, (ii) call rate<95%, or (iii) genotyping concordance rate<95% in quality control samples. The final dataset included 2,918 cases and 2,324 controls for 690,947 markers. There are 21,223 SNPs that were on Affymetrix 500K Array Set but not on the Affymetrix SNP Array 6.0. These SNPs were excluded. SNPs on the Affymetrix 6.0 array but not on the Affymetrix 500k array were treated as missing data for those samples genotyped on using the Affymetrix 500k array. Similar results were obtained after excluding women genotyped by Affymetrix 500K Array Set from the analyses.

Genotyping for Stage IIa was completed using the Illumina iSelect platform. To compare the consistency between the Affymetrix and Illumina iSelect platforms, we also included 43 samples from Stage I that were genotyped by Affymetrix SNP 6.0. Similar to the QC procedures used in Stage I, the following criteria were used to exclude samples: (i) call rate<95%; or (ii) unexpected duplicated samples based on IBD estimate. SNPs were excluded if: (i) call rate<95%, or (ii) genotyping concordance rate<95% in quality control samples when compared with Affymetrix 6.0 data. After QC, the mean concordance rate was 99.85% between Illumina iSelect and Affymetrix 6.0 genotyping.

Data for the SNPs analyzed in Stage IIb were extracted from the Korean GWAS genotyped using the Affymetrix Genome-Wide Human SNP Array 6.0 chip. A total of 30 QC samples were successfully genotyped, and the concordance rate was 99.83%. The sex of all samples was confirmed to be female. The SNPs were excluded if: (1) genotype call rate<95%, (2) MAF<1% in either the cases or controls, (3) deviation from HWE at *P*-value<10^−6^, and (4) poor cluster plot in either the cases or controls.

Genotyping for Stage III and all samples from Koreans in Stage IV was completed using the iPLEX Sequenom MassArray platform in the Vanderbilt Molecular Epidemiology Laboratory. Included in each 96-well plate as QC samples were one negative control (water), two blinded duplicates, and two samples from the HapMap project. To compare the consistency between the Affymetrix and Sequenom platforms, we also genotyped 45 samples included in Stage I. The mean concordance rate was 99.67% for the blind duplicates, 98.88% for HapMap samples, and 99.52% between Sequenom and Affymetrix 6.0 genotyping. Data quality from the Hong Kong study was low and thus data from the study were excluded for the current analysis. Genotyping for two Chinese studies (Nanjing and Guangzhou) in Stage IV was completed using the iPLEX Sequenom MassArray platform at the Fudan University, Shanghai, China. Blind duplicate QC samples were included and the mean concordance rate was 98.70%. Genotyping for the Tianjin study in Stage IV was performed using TaqMan assays. Genotyping assay protocols were developed and validated at the Vanderbilt Molecular Epidemiology Laboratory, and TaqMan genotyping assay reagents were provided to investigators of the Tianjin study (Tianjin Cancer Institute and Hospital). For the MEC study, data for the three SNPs presented in this study were extracted from the GWA scan data generated using Illumina 660W. For SNPs not included on the chip, imputed data using HapMap as reference were extracted. Genotype frequencies for SNP rs9485372 deviated from HWE in controls (P = 0.004), therefore, this SNP was excluded in data analyses. Not all SNPs for Stage IV were genotyped in all studies included in Stage IV due to genotyping failure or the use of different genotyping platforms (Table S8).

### SNP selection for replication

SNP selection for Stage II replication: Promising SNPs were selected for replication in Stage II based on the following criteria: 1) minor allele frequency (MAF)≥5%; 2) *P*<0.02 in Stage I; 3) Hardy-Weinberg equilibrium (HWE) test *P*>1.0×10^−6^ in controls; 4) not in strong linkage disequilibrium (LD) (r^2^<0.5) with any of the previously confirmed breast cancer genetic risk variants or SNPs evaluated in our previous studies [8], [12]; and 5) high genotyping quality as indicated by very clear genotyping clusters checked manually. When multiple SNPs are in LD with r^2^≥0.5, one SNP with the lowest *P*-value was selected. In total, 6,303 SNPs were selected for replication. A total of 5,906 SNPs (93.7%) were successfully designed by Illumina and included in the iSelect array. After stringent QC procedures, data from 5,365 SNPs were considered high quality for association analyses in Stage IIa, which include 1,613 breast cancer patients and 1,800 controls recruited from Shanghai studies.

SNP selection for Stage III replication: Among the 5,365 SNPs successfully genotyped in Stage IIa, 68 SNPs were selected for Stage III replication in an independent set of 5,203 cases and 5,138 controls recruited from Shanghai and several other East Asian populations (Table 1 and Text S1). The selection criteria are: 1) an association with breast cancer risk in Stage IIa with P≤0.05; 2) the direction of the association consistent in both stages; and 3) P≤0.001 in the merged data of Stage I and IIa. During the course of Stage III genotyping, genome-wide association scan data from 2,359 cases and 2,052 controls were obtained from the Seoul Breast Cancer GWAS (Stage IIb). Therefore, we performed a meta-analysis of Stage IIa and IIb data. Of the 5,297 SNPs which were not selected initially for Stage III replication based on Stage IIa data alone, data were available for 4,913 SNPs in Stage IIb. Meta-analyses of these 4,913 SNPs from Stage IIa and IIb yielded 26 additional SNPs that showed an association at *P*≤0.05 and in the same direction among stages I, IIa, and IIb. These 26 SNPs were then added to the list of SNPs to be genotyped in Stage III.

SNP selection for Stage IV replication: Based on the results of the first three stages, 22 top SNPs were selected for Stage IV evaluation and genotyped in up to 17,423 additional subjects (7,489 cases and 9,934 controls) (Table 1 and Text S1).

### Statistical analyses

Case-control differences in selected demographic characteristics and major risk factors were evaluated using t-tests (for continuous variables) and Chi-square tests (for categorical variables). Associations between SNPs and breast cancer risk were assessed using odds ratios (ORs) and 95% confidence intervals (CIs) derived from logistic regression models. ORs were estimated for heterozygote and homozygote for the variant allele compared with homozygotes for the common allele. ORs were also estimated for the variant allele based on a log-additive model and adjusted for age, and study site, when appropriate. Stratified analyses by ethnicity, menopausal status, and estrogen receptor (ER) status were carried out. PLINK version 1.06 was used to analyze genome-wide data obtained in Stage I and the replication data in Stage IIa. Results from Stage IIb were also obtained from PLINK version 1.06. Meta-analyses of Stage IIa and Stage IIb were performed using a weighted z-statistics method, where weights were proportional to the square root of the number of individuals in each sample and standardized such that the weights added up to one. The z-statistic summarizes the magnitude and direction of the effect relative to the reference allele. An overall z-statistic and p value were then calculated from the weighted average of the individual statistics. Calculations were implemented in the METAL package (http://www.sph.umich.edu/csg/abecasis/Metal). Individual data were obtained from each study for Stage IV SNPs for a pooled analysis, which were conducted using SAS, version 9.2, with the use of two-tailed tests.

**Figure 4:**
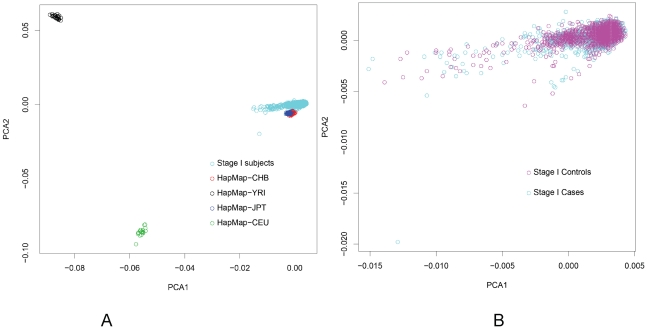
Principal Component Analysis (PCA) based on the first two eigenvectors obtained by PCA. A: all individuals from Stage I and HapMap; B: breast cancer cases and controls from Stage I.

We first investigated the population structure by estimating inflation factor λ using all 690,947 SNPs SNPs that passed the QC. The inflation factor λ was estimated to be 1.042, suggesting that any population substructure, if present, should not have any appreciable effect on the results. Among the final 690,947 SNPs obtained in Stage I after QC, we generated a list of 196,471 SNPs with pairwise LD<0.2 by using plink (http://pngu.mgh.harvard.edu/~purcell/plink/). Then, principal components were estimated based on these 196,471 SNPs using EIGENSTRAT [36]. We then drew a plot for all Stage I and HapMap II subjects based on the first two principal components (Figure 4). All study participants in Stage I were clustered very closely with HapMap Asians. The first 5 or 10 principal components were adjusted in the logistic regression analyses for evaluating associations of SNPs and breast cancer risk.

To evaluate the combined effect of SNPs located in chromosome 6q25.1 on breast cancer risk, we created a genetic risk score (GRS) by summing the number (0–2) of risk alleles that each woman carried for each of the three SNPs, including rs9383951, rs9485372, rs2046210. The GRS was constructed among those who had complete data for all three SNPs. We also did imputation using MACH (http://www.sph.umich.edu/csg/abecasis/MACH/index.html) with HapMap II Asian data as reference. LD structure was estimated from the flanking 100 kb of these three SNPs and the *ESR1* gene using data from HapMap II Asians (Figure S1). All SNPs in the LD blocks including rs9485372, rs2046210 and rs9383951 and SNPs inside the *ESR1* gene were analyzed in relation to breast cancer risk with age, rs9485372, rs9383951 and rs2046210 adjusted.

## Supporting Information

Figure S1 Estimates of pairwise LD (r^2^) for common SNPs from HapMap II Asians for the SNPs located in 6q25.1. A: LD plot for the flanking 100 kb of SNP rs9485372. B: LD plot for the upstream 100 kb of SNP rs2046210 and the ESR1 gene.
(TIF)

Figure S2 Estimates of pairwise LD (r^2^) from HapMap II Asian for the SNPs showing significant associations after adjusted for rs9485372, rs9383951 and rs2046210.
(TIF)

Figure S3 A regional plot of the −log_10_P-values for SNPs at 11q24.3. The LD is estimated using data from HapMap Asian population. Also shown are the SNP Build 36 coordinates in kilobases (Kb), recombination rates in centimorgans (cM) per megabase (Mb) and genes in the region (below) based on the March 2006 UCSC genome browser assembly.
(TIF)

Table S1 Association of SNPs with breast cancer risk by menopause and ER status.
(DOCX)

Table S2 Association results adjusted for the top principal components in Stage I.
(DOCX)

Table S3 LD between the 3 SNPs that are associated with breast cancer and are located in 6q25.1.
(DOCX)

Table S4 Conditional analyses for SNPs located on 6q25.1.
(DOCX)

Table S5 Association results of SNP-SNP interaction.
(DOCX)

Table S6 Associations of breast cancer risk with the genetic risk score for the three SNPs located in chromosome 6q25.1, the Asia Breast Cancer Consortium.
(DOCX)

Table S7 SNPs in 6q25.1 showed association after adjusted for rs9485372, rs9383951 and rs2046210.
(DOCX)

Table S8 Sample size for the SNPs included in Stage IV.
(DOCX)

Text S1 Supplementary Methods.
(DOCX)
