# Supplementary figures and images for: Genome-Wide Association Study in East Asians Identifies Novel Susceptibility Loci for Breast Cancer
Source: PLoS Genet. 2012 Feb 23;8(2):e1002532. doi: 10.1371/journal.pgen.1002532 (PMC3285588; doi:10.1371/journal.pgen.1002532)

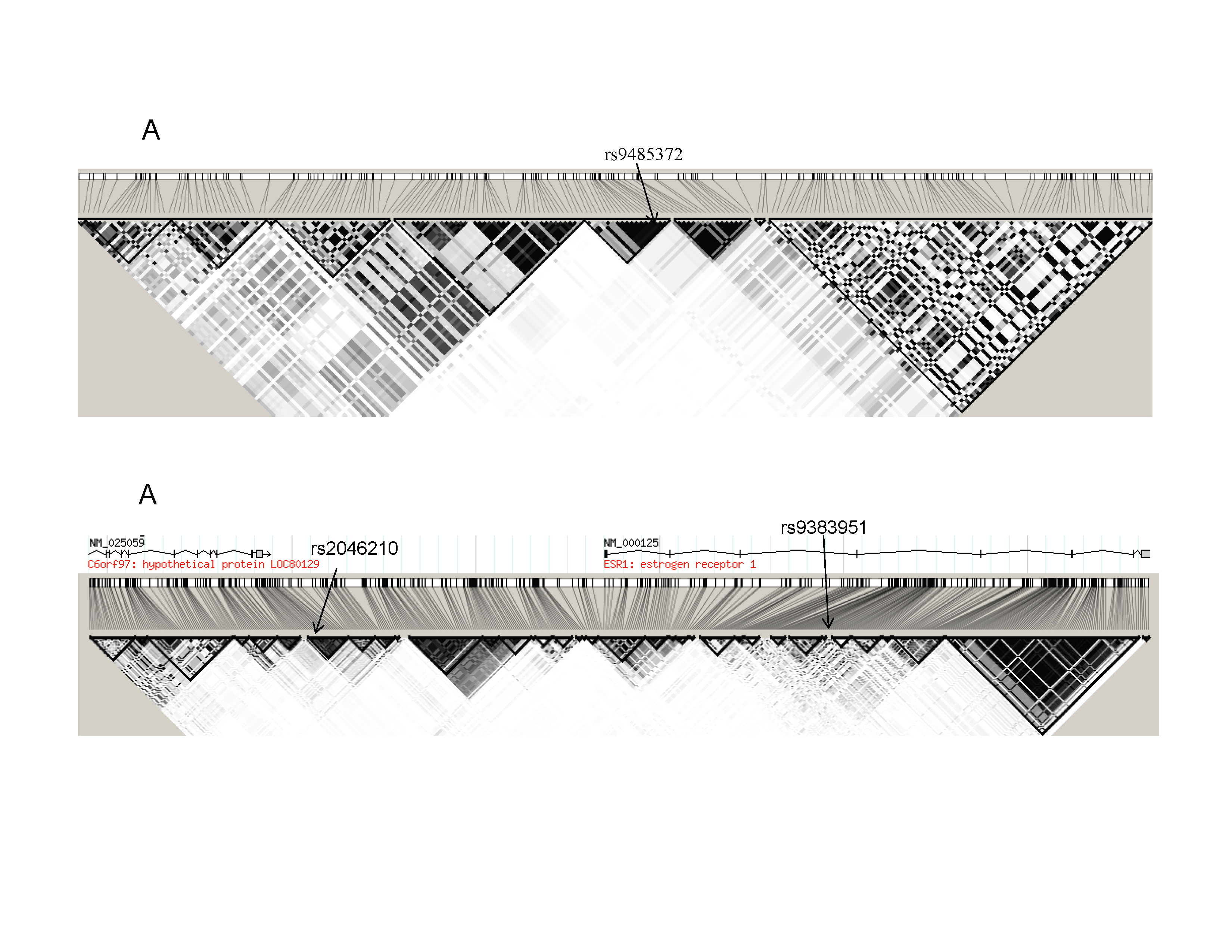

Supplement: Figure S1 — Estimates of pairwise LD (r2) for common SNPs from HapMap II Asians for the SNPs located in 6q25.1. A: LD plot for the flanking 100 kb of SNP rs9485372. B: LD plot for the upstream 100 kb of SNP rs2046210 and the ESR1 gene. (TIF) [file pgen.1002532.s001.tif]

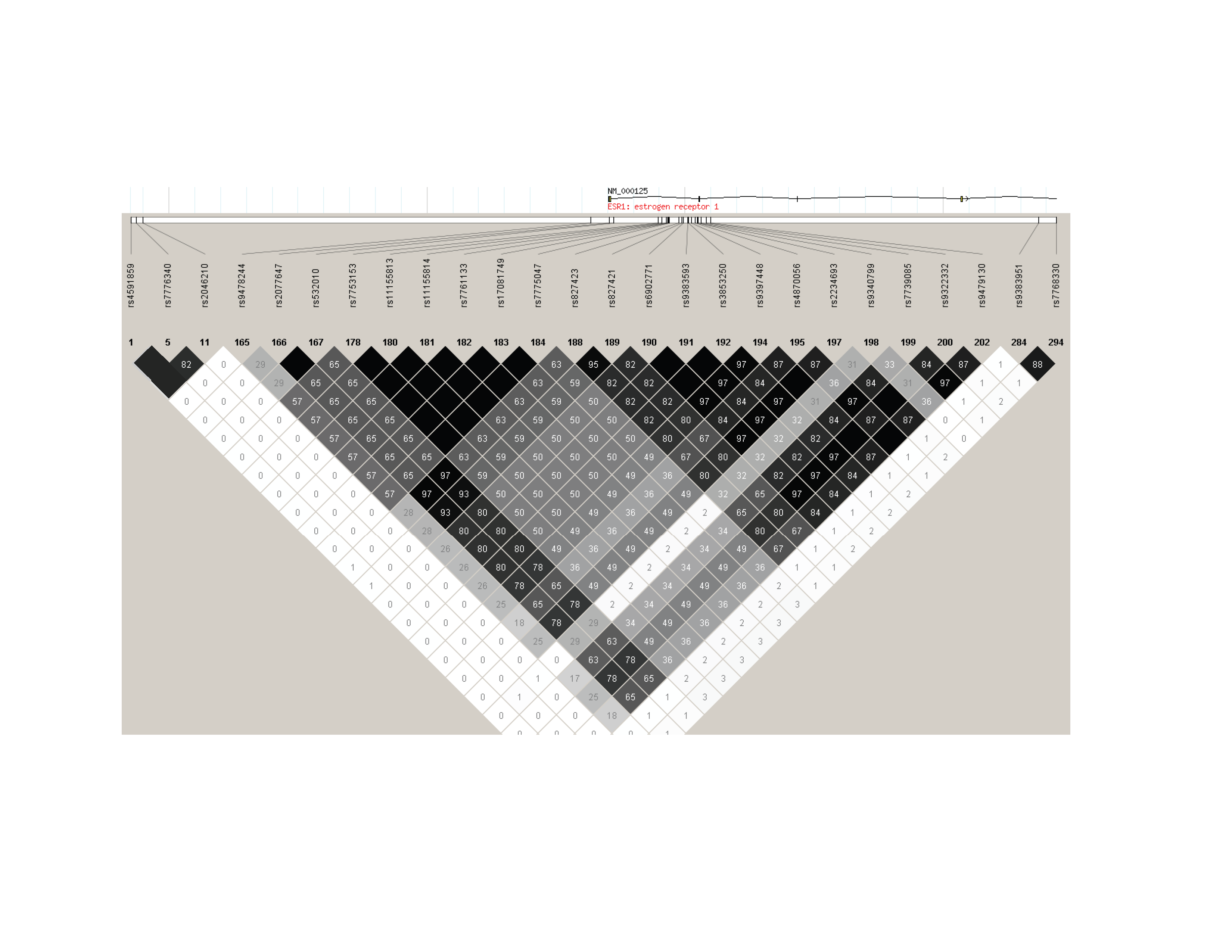

Supplement: Figure S2 — Estimates of pairwise LD (r2) from HapMap II Asian for the SNPs showing significant associations after adjusted for rs9485372, rs9383951 and rs2046210. (TIF) [file pgen.1002532.s002.tif]

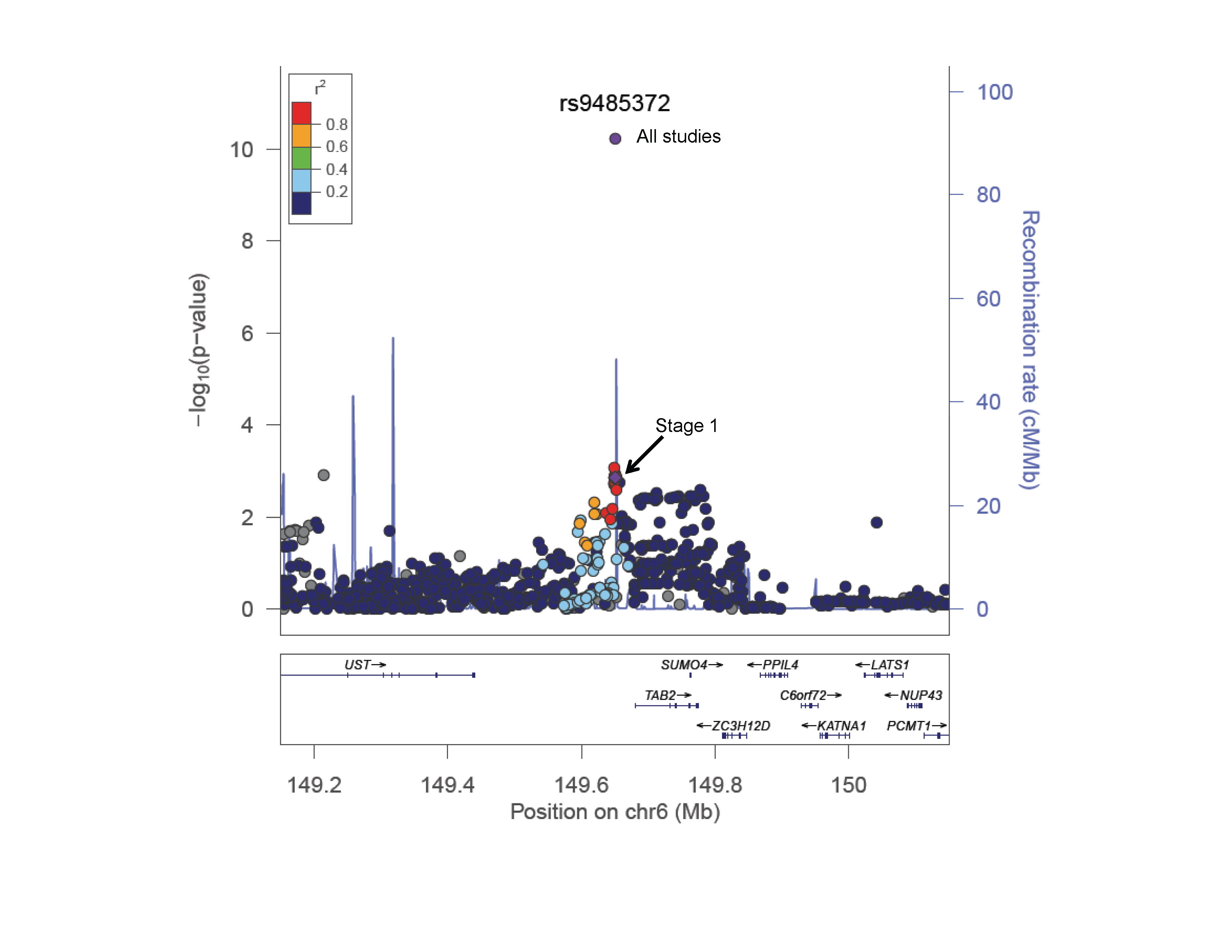

Supplement: Figure S3 — A regional plot of the −log10P-values for SNPs at 11q24.3. The LD is estimated using data from HapMap Asian population. Also shown are the SNP Build 36 coordinates in kilobases (Kb), recombination rates in centimorgans (cM) per megabase (Mb) and genes in the region (below) based on the March 2006 UCSC genome browser assembly. (TIF) [file pgen.1002532.s003.tif]
