# Supplementary material for: Genome-Wide Association Study in East Asians Identifies Novel Susceptibility Loci for Breast Cancer
Source: PLoS Genet. 2012 Feb 23;8(2):e1002532. doi: 10.1371/journal.pgen.1002532 (PMC3285588; doi:10.1371/journal.pgen.1002532)
Supplement: Table S1 — Association of SNPs with breast cancer risk by menopause and ER status. (DOCX) [file pgen.1002532.s004.docx]

| **Table S1.** Association of SNPs with breast cancer risk by menopause and ER status^a^ | | | | | |  |  |
| --- | --- | --- | --- | --- | --- | --- | --- |
| SNP | Group | No. of Cases/ No. of Controls | EAF (%)^b^ | OR (95% CI)^c^ |  | P_for trend_^c^ | P_heterogeneity_^c^ |
|  |  |  |  | Heterozygote | Homozygote |  |  |
| rs9485372 |  |  |  |  |  |  |  |
|  | ER positive | 6917/12262 | 44.05 | 0.91(0.85-0.97) | 0.82(0.75-0.89) | 3.7 × 10^-6^ | 0.34 |
|  | ER negative | 3826/12262 | 44.05 | 0.90(0.83-0.98) | 0.89(0.80-0.99) | 0.016 |  |
|  | PR positive | 6329/12262 | 44.05 | 0.92(0.86-0.98) | 0.83(0.76-0.90) | 2.5 × 10^-5^ | 0.49 |
|  | PR negative | 4201/12262 | 44.05 | 0.89(0.82-0.96) | 0.88(0.79-0.97) | 4.9 × 10^-3^ |  |
|  | Pre-menopausal | 6302/6073 | 44.05 | 0.90(0.83-0.98) | 0.82(0.74-0.91) | 1.6 × 10^-4^ | 0.83 |
|  | Post-menopausal | 5751/5594 | 43.7 | 0.91(0.83-0.99) | 0.85(0.76-0.95) | 1.8 × 10^-3^ |  |
|  | BM≥median | 4086/3645 | 43.15 | 0.92(0.83-1.01) | 0.84(0.74-0.96) | 9.3 × 10^-3^ | 0.87 |
|  | BM<median | 4322/4349 | 43.6 | 0.91(0.83-1.01) | 0.83(0.73-0.94) | 2.3 × 10^-3^ |  |
|  | WHR≥median | 3686/2634 | 42.75 | 0.99(0.89-1.11) | 0.87(0.75-1.01) | 0.10 | 0.29 |
|  | WHR<median | 2664/3325 | 43.35 | 0.81(0.72-0.91) | 0.82(0.71-0.96) | 2.3 × 10^-3^ |  |
|  | Menstruation≥median | 3567/3151 | 43.65 | 0.88(0.79-0.98) | 0.85(0.74-0.98) | 0.014 | 0.76 |
|  | Menstruation<median | 2704/2851 | 44.4 | 0.93(0.82-1.05) | 0.79(0.68-0.92) | 3.9 × 10^-3^ |  |
| rs9383951 |  |  |  |  |  |  |  |
|  | ER positive | 7986/14261 | 10.05 | 0.82(0.76-0.89) | 0.86(0.65-1.13) | 7.8 × 10^-7^ | 0.55 |
|  | ER negative | 4160/14261 | 10.05 | 0.88(0.80-0.96) | 0.78(0.55-1.12) | 2.7 × 10^-3^ |  |
|  | PR positive | 7268/14261 | 10.05 | 0.84(0.78-0.91) | 0.84(0.63-1.11) | 1.3 × 10^-5^ | 0.87 |
|  | PR negative | 4638/14261 | 10.05 | 0.83(0.76-0.91) | 0.86(0.62-1.20) | 9.8 × 10^-5^ |  |
|  | Pre-menopausal | 6839/6467 | 10.25 | 0.88(0.81-0.97) | 0.84(0.60-1.18) | 5.2 × 10^-3^ | 0.32 |
|  | Post-menopausal | 6264/6010 | 10.45 | 0.83(0.75-0.91) | 0.71(0.51-1.00) | 1.6 × 10^-5^ |  |
|  | BM≥median | 4457/4000 | 10.55 | 0.87(0.78-0.97) | 0.91(0.60-1.38) | 0.02 | 0.42 |
|  | BM<median | 4688/4742 | 10.2 | 0.84(0.75-0.94) | 0.72(0.49-1.07) | 5.8 × 10^-4^ |  |
|  | WHR≥median | 4009/2839 | 11.25 | 0.80(0.71-0.91) | 0.95(0.61-1.47) | 2.2 × 10^-3^ | 0.85 |
|  | WHR<median | 2989/3602 | 10.95 | 0.84(0.74-0.95) | 0.86(0.54-1.36) | 7.3 × 10^-3^ |  |
|  | Menstruation≥median | 3899/3399 | 10.65 | 0.91(0.80-1.02) | 0.76(0.49-1.17) | 0.049 | 0.96 |
|  | Menstruation<median | 2897/2991 | 9.7 | 0.87(0.76-1.00) | 1.09(0.63-1.88) | 0.10 |  |
| rs7107217 |  |  |  |  |  |  |  |
|  | ER positive | 8353/15172 | 34.85 | 1.07(1.01-1.13) | 1.11(1.01-1.21) | 7.3 × 10^-3^ | 0.71 |
|  | ER negative | 4762/15172 | 34.85 | 1.08(1.01-1.16) | 1.16(1.05-1.29) | 2.0 × 10^-3^ |  |
|  | PR positive | 7669/15172 | 34.85 | 1.06(1.00-1.12) | 1.11(1.01-1.21) | 0.01 | 0.61 |
|  | PR negative | 5218/15172 | 34.85 | 1.09(1.01-1.16) | 1.15(1.04-1.27) | 2.2 × 10^-3^ |  |
|  | Pre-menopausal | 7285/6971 | 33.75 | 1.10(1.02-1.18) | 1.09(0.98-1.21) | 0.02 | 0.45 |
|  | Post-menopausal | 6722/6750 | 33.85 | 1.08(1.00-1.16) | 1.22(1.09-1.36) | 4.0 × 10^-4^ |  |
|  | BM≥median | 5181/4984 | 33.4 | 1.08(1.00-1.18) | 1.24(1.09-1.41) | 8.3 × 10^-4^ | 0.82 |
|  | BM<median | 5132/5046 | 33.85 | 1.11(1.02-1.20) | 1.16(1.02-1.32) | 5.6 × 10^-3^ |  |
|  | WHR≥median | 4028/2846 | 31.05 | 1.11(1.00-1.23) | 1.36(1.15-1.61) | 2.5 × 10^-4^ | 0.14 |
|  | WHR<median | 2997/3614 | 32.5 | 1.07(0.96-1.18) | 1.11(0.94-1.31) | 0.14 |  |
|  | Menstruation≥median | 4151/3576 | 33.35 | 1.08(0.98-1.18) | 1.13(0.97-1.31) | 0.06 | 0.91 |
|  | Menstruation<median | 3101/3323 | 34.1 | 1.13(1.02-1.26) | 1.05(0.90-1.24) | 0.14 |  |
| ^a^ SeBCS-I and KOHBRA/KoGES were not included in stratified analyses for ER and menopause status | | | | | | | |
| ^b^ Effect allele frequency in controls. | | | | | | | |
| ^c^ Adjusted for age and study sites. | | | | | | | |
