# Supplementary material for: Genome-Wide Association Study in East Asians Identifies Novel Susceptibility Loci for Breast Cancer
Source: PLoS Genet. 2012 Feb 23;8(2):e1002532. doi: 10.1371/journal.pgen.1002532 (PMC3285588; doi:10.1371/journal.pgen.1002532)
Supplement: Table S2 — Association results adjusted for the top principal components in Stage I. (DOCX) [file pgen.1002532.s005.docx]

| Table S2 Association results adjusted for the top principal components in Stage I^a^ | | | | | | | | | |
| --- | --- | --- | --- | --- | --- | --- | --- | --- | --- |
| SNP | Not adjusted for PCs | | | Adjusted for top 5 PCs | | | Adjusted for top 10 PCs | | |
|  | OR (95% CI) | | P_trend_ | OR (95% CI) | | P_trend_ | OR (95% CI) | | P_trend_ |
|  | Heterozyte | Homozygote |  | Heterozyte | Homozygote |  | Heterozyte | Homozygote |  |
| rs9485372 | 0.82(0.72-0.93) | 0.79(0.67-0.93) | 1.4 × 10^-3^ | 0.82(0.72-0.93) | 0.78(0.66-0.93) | 1.2 × 10^-3^ | 0.82(0.72-0.93) | 0.79(0.66-0.93) | 1.3 × 10^-3^ |
| rs9383951 | 0.80(0.69-0.92) | 0.82(0.51-1.34) | 2.4 × 10^-3^ | 0.80(0.69-0.92) | 0.83(0.51-1.35) | 2.6 × 10^-3^ | 0.80(0.69-0.92) | 0.83(0.51-1.34) | 2.2 × 10^-3^ |
| rs7107217 | 1.13(1.01-1.27) | 1.27(1.06-1.53) | 3.6 × 10^-3^ | 1.13(1.00-1.27) | 1.27(1.05-1.53) | 4.5 × 10^-3^ | 1.13(1.00-1.27) | 1.27(1.06-1.53) | 4.3 × 10^-3^ |
| ^a^ Among 2,918 cases and 2,324 controls included in Stage I | | | | |  |  |  |  |  |
