# Supplementary material for: Genome-Wide Association Study in East Asians Identifies Novel Susceptibility Loci for Breast Cancer
Source: PLoS Genet. 2012 Feb 23;8(2):e1002532. doi: 10.1371/journal.pgen.1002532 (PMC3285588; doi:10.1371/journal.pgen.1002532)
Supplement: Table S3 — LD between the 3 SNPs that are associated with breast cancer and are located in 6q25.1. (DOCX) [file pgen.1002532.s006.docx]

| Table S3 LD between the 3 SNPs that are associated with breast cancer and are located in 6q25.1 | | | | |
| --- | --- | --- | --- | --- |
| Study | population | rs9485372 vs rs2046210 | rs9383951 vs rs2046210 | rs9485372 vs rs9383951 |
| HapMap | CHBJPT | 0.01 | 0.04 | 0.08 |
|  | CEU | 0.11 | 0.05 | 0.06 |
|  | YRI | 0.02 | 0.09 | 0.01 |
| Present Study | Chinese | 0.003 | 0.02 | 0.003 |
|  | Japanese | 0.005 | 0.01 | 0.02 |
|  | Korean | 0.02 | 0.02 | 0.008 |
|  |  |  |  |  |
