# Supplementary material for: Genome-Wide Association Study in East Asians Identifies Novel Susceptibility Loci for Breast Cancer
Source: PLoS Genet. 2012 Feb 23;8(2):e1002532. doi: 10.1371/journal.pgen.1002532 (PMC3285588; doi:10.1371/journal.pgen.1002532)
Supplement: Table S4 — Conditional analyses for SNPs located on 6q25.1. (DOCX) [file pgen.1002532.s007.docx]

| Table S4 Conditional analyses for SNPs located on 6q25.1^a^ | | | |  |  |
| --- | --- | --- | --- | --- | --- |
| SNP | SNP adjusted | OR^b^ | | | P^b^ |
|  |  | Heterozygote | Homozygote | Per allele |  |
| rs9485372 | no adjustment | 0.87(0.83-0.92) | 0.79(0.73-0.84) | 0.88(0.86-0.92) | 9.2 x 10^-13^ |
| rs9485372 | rs2046210 | 0.87(0.83-0.92) | 0.79(0.74-0.84) | 0.89(0.86-0.92) | 1.5 x 10^-12^ |
| rs9485372 | rs9383951 | 0.87(0.83-0.92) | 0.79(0.73-0.84) | 0.88(0.86-0.91) | 8.2 x 10^-13^ |
| rs9485372 | rs2046210 and rs9383951 | 0.87(0.83-0.92) | 0.79(0.74-0.84) | 0.89(0.86-0.92) | 1.4 x 10^-12^ |
|  |  |  |  |  |  |
| rs9383951 | no adjustment | 0.88(0.83-0.94) | 0.92(0.72-1.16) | 0.90(0.85-0.95) | 1.5 x 10^-4^ |
| rs9383951 | rs2046210 | 0.88(0.83-0.94) | 0.92(0.73-1.16) | 0.90(0.85-0.95) | 1.5 x 10^-4^ |
| rs9383951 | rs9485372 | 0.88(0.83-0.94) | 0.91(0.72-1.15) | 0.90(0.85-0.95) | 1.3 x 10^-4^ |
| rs9383951 | rs2046210 and rs9485372 | 0.88(0.83-0.94) | 0.92(0.72-1.16) | 0.90(0.85-0.95) | 1.3 x 10^-4^ |
|  |  |  |  |  |  |
| rs2046210 | no adjustment | 1.28(1.21-1.35) | 1.57(1.46-1.69) | 1.26(1.22-1.30) | 3.6 x 10^-39^ |
| rs2046210 | rs9485372 | 1.28(1.21-1.35) | 1.57(1.46-1.69) | 1.26(1.22-1.30) | 5.8 x 10^-39^ |
| rs2046210 | rs9383951 | 1.28(1.21-1.35) | 1.57(1.46-1.69) | 1.26(1.22-1.30) | 3.7 x 10^-39^ |
| rs2046210 | rs9485372 and rs9383951 | 1.28(1.21-1.35) | 1.57(1.46-1.69) | 1.26(1.22-1.30) | 6.0 x 10^-39^ |
| ^a^ Among 15105 cases and 15048 controls with genotype data available for all three SNPs. | | | | |  |
| ^b^ Adjusted for age and study center | |  |  |  |  |
|  |  |  |  |  |  |
