# Supplementary material for: Genome-Wide Association Study in East Asians Identifies Novel Susceptibility Loci for Breast Cancer
Source: PLoS Genet. 2012 Feb 23;8(2):e1002532. doi: 10.1371/journal.pgen.1002532 (PMC3285588; doi:10.1371/journal.pgen.1002532)
Supplement: Table S5 — Association results of SNP-SNP interaction. (DOCX) [file pgen.1002532.s008.docx]

| Table S5 Association results of SNP-SNP interaction | | | | | | | |
| --- | --- | --- | --- | --- | --- | --- | --- |
| SNP | No. of cases/controls | OR (95% CI) | No. of cases/controls | OR(95% CI) | No. of cases/controls | OR (95% CI)^a^ | P_interaction_ |
|  |  |  |  |  |  |  |  |
| rs2046210 | | | | | | | |
| rs9485372 | G/G | | A/G | | A/A | |  |
| A/A | 1003/1332 | 1.00 (ref) | 1288/1440 | 1.15(1.03-1.29) | 425/356 | 1.51(1.27-1.79) | 0.20 |
| A/G | 2600/3212 | 1.05(0.95-1.16) | 3634/3409 | 1.36(1.23-1.50) | 1183/901 | 1.64(1.45-1.85) |  |
| G/G | 1745/1916 | 1.17(1.05-1.31) | 2488/1985 | 1.58(1.42-1.75) | 816/533 | 1.90(1.65-2.19) |  |
|  |  |  |  |  |  |  |  |
| rs2046210 | | | | | | | |
| rs9383951 | G/G | | A/G | | A/A | |  |
| C/C | 49/89 | 1.00 (ref) | 80/67 | 2.33(1.42-3.82) | 28/18 | 3.03(1.48-6.20) | 0.13 |
| C/G | 959/1277 | 1.45(1.00-2.11) | 1342/1365 | 1.84(1.27-2.66) | 435/357 | 2.41(1.63-3.57) |  |
| G/G | 5082/5922 | 1.68(1.17-2.43) | 6807/6161 | 2.15(1.49-3.09) | 2215/1608 | 2.60(1.80-3.77) |  |
|  |  |  |  |  |  |  |  |
| rs2046210 | | | | | | | |
| rs7107217 | G/G | | A/G | | A/A | |  |
| A/A | 2500/3172 | 1.00 (ref) | 3494/3365 | 1.29(1.20-1.39) | 1166/875 | 1.65(1.48-1.83) | 0.78 |
| A/C | 3011/3456 | 1.14(1.06-1.23) | 3956/3579 | 1.43(1.33-1.54) | 1284/951 | 1.73(1.56-1.92) |  |
| C/C | 868/1050 | 1.10(0.99-1.23) | 1207/1063 | 1.53(1.38-1.69) | 366/266 | 1.77(1.49-2.11) |  |
|  |  |  |  |  |  |  |  |
| rs9383951 | | | | | | | |
| rs9485372 | C/C | | C/G | | G/G | |  |
| A/A | 29/36 | 1.00 (ref) | 475/628 | 0.91(0.54-1.51) | 2353/2927 | 0.97(0.59-1.60) | 0.09 |
| A/G | 71/80 | 1.03(0.57-1.86) | 1277/1541 | 0.98(0.59-1.63) | 6402/7025 | 1.08(0.66-1.78) |  |
| G/G | 48/68 | 0.84(0.45-1.57) | 851/954 | 1.05(0.63-1.74) | 4373/4184 | 1.24(0.75-2.04) |  |
|  |  |  |  |  |  |  |  |
| rs7107217 | | | | | | | |
| rs9485372 | A/A | | A/C | | C/C | |  |
| A/A | 1144/1546 | 1.00 (ref) | 1296/1615 | 1.12(1.00-1.24) | 413/432 | 1.33(1.13-1.56) | 0.06 |
| A/G | 3115/3606 | 1.15(1.05-1.26) | 3593/3923 | 1.25(1.14-1.37) | 1044/1111 | 1.31(1.17-1.47) |  |
| G/G | 2133/2178 | 1.29(1.17-1.43) | 2449/2311 | 1.44(1.30-1.58) | 686/716 | 1.34(1.17-1.52) |  |
|  |  |  |  |  |  |  |  |
| rs9383951 | | | | | | | |
| rs7107217 | C/C | | C/G | | G/G | |  |
| A/A | 68/90 | 1.00 (ref) | 1114/1405 | 1.05(0.75-1.46) | 5755/6295 | 1.22(0.88-1.68) | 0.97 |
| A/C | 65/88 | 1.01(0.64-1.60) | 1347/1494 | 1.22(0.88-1.70) | 6669/6916 | 1.32(0.95-1.82) |  |
| C/C | 24/19 | 1.66(0.84-3.31) | 347/442 | 1.09(0.77-1.55) | 2060/2043 | 1.40(1.01-1.93) |  |
| ^a^ Adjusted for age and study center | | | | | | | |
