# Supplementary material for: Genome-Wide Association Study in East Asians Identifies Novel Susceptibility Loci for Breast Cancer
Source: PLoS Genet. 2012 Feb 23;8(2):e1002532. doi: 10.1371/journal.pgen.1002532 (PMC3285588; doi:10.1371/journal.pgen.1002532)
Supplement: Table S6 — Associations of breast cancer risk with the genetic risk score for the three SNPs located in chromosome 6q25.1, the Asia Breast Cancer Consortium. (DOCX) [file pgen.1002532.s009.docx]

| Table S6. Associations of breast cancer risk with the genetic risk score for the three SNPs located in chromosome 6q25.1, the Asia Breast Cancer Consortiuma^a^ | | | |
| --- | --- | --- | --- |
| No. of risk variants | No. of cases | No. of controls | OR (95% CI)^b^ |
| <=1 | 198 | 303 | 1.00 (reference) |
| 2 | 1,567 | 1,920 | 1.32 (1.08-1.61) |
| 3 | 4,126 | 4,890 | 1.34 (1.10-1.62) |
| 4 | 5,403 | 5,083 | 1.67 (1.38-2.02) |
| 5 | 3,136 | 2,419 | 2.00 (1.65-2.44) |
| 6 | 675 | 433 | 2.36 (1.89-2.96) |
| P _for trend_ |  |  | 1.3 x 10^-47^ |
| ^a^ Among women with genotyping data for all three SNPs, rs9485372, rs9383951 and rs2046210. | | | |
| ^b^ Adjusted for age and study site | | |  |
