# Supplementary material for: Genome-Wide Association Study in East Asians Identifies Novel Susceptibility Loci for Breast Cancer
Source: PLoS Genet. 2012 Feb 23;8(2):e1002532. doi: 10.1371/journal.pgen.1002532 (PMC3285588; doi:10.1371/journal.pgen.1002532)
Supplement: Table S7 — SNPs in 6q25.1 showed association after adjusted for rs9485372, rs9383951 and rs2046210. (DOCX) [file pgen.1002532.s010.docx]

| Table S7 SNPs in 6q25.1 showed association after adjusted for rs9485372, rs9383951 and rs2046210 | | | | | | | | |
| --- | --- | --- | --- | --- | --- | --- | --- | --- |
| SNP | Position^a^ | Alleles^b^ | EAF (%)^c^ | OR (95% CI)^d^ | P^d^ | OR (95% CI)^e^ | P^e^ | RSQ |
| rs4591859 | 151985332 | C/T | 43.1 | 1.27(1.17-1.37) | 8.6 × 10^-9^ | 1.18(1.02-1.36) | 0.03 | 0.94 |
| rs7776340 | 151987359 | C/T | 57.8 | 0.80(0.73-0.86) | 1.4 × 10^-8^ | 0.86(0.74-0.99) | 0.04 | 0.99 |
| rs2077647 | 152170770 | C/T | 37.2 | 0.92(0.85-1.00) | 0.05 | 0.91(0.83-0.99) | 0.02 | 0.94 |
| rs532010 | 152172611 | A/G | 62.9 | 1.09(1.00-1.18) | 0.05 | 1.10(1.01-1.20) | 0.02 | 0.94 |
| rs9371227 | 152175310 | A/C | 26.4 | 1.11(1.02-1.21) | 0.02 | 1.14(1.05-1.25) | 3.2 × 10^-3^ | 0.98 |
| rs17081713 | 152176440 | A/G | 85.5 | 1.13(1.00-1.28) | 0.06 | 1.15(1.02-1.31) | 0.03 | 0.79 |
| rs9371557 | 152181902 | A/G | 73.7 | 0.90(0.82-0.98) | 0.02 | 0.88(0.80-0.96) | 3.3 × 10^-3^ | 1.00 |
| rs7753153 | 152189791 | A/G | 27.5 | 0.93(0.85-1.01) | 0.10 | 0.91(0.84-1.00) | 0.05 | 1.00 |
| rs11155813 | 152191128 | C/T | 27.5 | 0.93(0.85-1.01) | 0.09 | 0.91(0.84-1.00) | 0.05 | 1.00 |
| rs12665044 | 152191565 | C/T | 72.5 | 1.08(0.99-1.18) | 0.09 | 1.09(1.00-1.20) | 0.05 | 0.99 |
| rs11155814 | 152192877 | A/G | 72.5 | 1.08(0.99-1.18) | 0.09 | 1.09(1.00-1.20) | 0.05 | 0.99 |
| rs7761133 | 152193556 | C/T | 27.5 | 0.93(0.85-1.01) | 0.09 | 0.91(0.84-1.00) | 0.05 | 0.99 |
| rs17081749 | 152193618 | G/T | 27.5 | 0.93(0.85-1.01) | 0.09 | 0.91(0.84-1.00) | 0.05 | 0.99 |
| rs7775047 | 152193886 | C/G | 27.5 | 0.93(0.85-1.01) | 0.09 | 0.91(0.84-1.00) | 0.05 | 0.99 |
| rs827423 | 152197890 | A/G | 63.0 | 1.09(1.01-1.19) | 0.03 | 1.11(1.02-1.20) | 0.02 | 0.99 |
| rs827421 | 152198815 | A/G | 63.0 | 1.10(1.01-1.19) | 0.04 | 1.11(1.02-1.22) | 0.01 | 0.90 |
| rs6902771 | 152199574 | C/T | 60.4 | 1.09(1.01-1.19) | 0.03 | 1.11(1.02-1.21) | 0.01 | 0.93 |
| rs9383593 | 152201319 | A/T | 60.4 | 1.09(1.01-1.19) | 0.03 | 1.11(1.02-1.21) | 0.01 | 0.94 |
| rs3853250 | 152201593 | G/T | 39.6 | 0.91(0.84-0.99) | 0.03 | 0.90(0.83-0.98) | 0.01 | 0.94 |
| rs9397448 | 152202759 | A/G | 39.8 | 0.92(0.84-0.99) | 0.04 | 0.90(0.83-0.98) | 0.01 | 0.96 |
| rs4870056 | 152203920 | A/G | 36.9 | 0.92(0.85-1.00) | 0.05 | 0.90(0.83-0.98) | 0.02 | 0.95 |
| rs2234693 | 152205028 | C/T | 39.9 | 0.92(0.85-0.99) | 0.04 | 0.90(0.83-0.98) | 0.01 | 0.98 |
| rs9340799 | 152205074 | A/G | 78.3 | 1.08(0.97-1.20) | 0.15 | 1.12(1.00-1.24) | 0.04 | 0.82 |
| rs7739085 | 152206241 | C/G | 35.7 | 0.91(0.84-1.00) | 0.04 | 0.90(0.83-0.98) | 0.02 | 0.92 |
| rs9322332 | 152208494 | A/C | 39.7 | 0.92(0.85-1.00) | 0.04 | 0.90(0.83-0.98) | 0.01 | 0.95 |
| rs9479130 | 152210149 | A/C | 63.3 | 1.09(1.00-1.18) | 0.05 | 1.11(1.02-1.21) | 0.02 | 0.92 |
| rs7768330 | 152343905 | A/G | 88.7 | 1.19(1.04-1.36) | 0.01 | 0.43(0.21-0.87) | 0.02 | 0.86 |
| ^a^ From NCBI genome build 36. | | | | | | | | |
| ^b^ Effect/reference alleles based on forward strand. | | | | | | | | |
| ^c^ Effect allele frequency in controls. | | | | | | | | |
| ^d^ Adjusted for age. | | | | | | | | |
| ^e^ Adjusted for age, rs9485372, rs9383951 and rs2046210. | | | | | | | | |
