# Supplementary material for: Genome-Wide Association Study in East Asians Identifies Novel Susceptibility Loci for Breast Cancer
Source: PLoS Genet. 2012 Feb 23;8(2):e1002532. doi: 10.1371/journal.pgen.1002532 (PMC3285588; doi:10.1371/journal.pgen.1002532)
Supplement: Table S8 — Sample size for the SNPs included in Stage IV. (DOCX) [file pgen.1002532.s011.docx]

| Table S8 Sample size for the SNPs included in Stage IV^a^ | | | | |  |  |
| --- | --- | --- | --- | --- | --- | --- |
| SNP | StageI | StageII | StageIII | StageIV | All | Study with genotype data missing |
| rs10107389 | 2904/2312 | 3927/3805 | 5027/5053 | 5204/7446 | 17062/18616 | MEC,Tianjin |
| rs10511591 | 2869/2274 | 1613/1800 | 4954/4980 | 2561/2662 | 11997/11716 | KOHBRA/KoGES,MEC,NCC,SeBCS-I,SeBCS-II,Tianjin |
| rs1078985 | 2909/2316 | 3884/3798 | 4991/5051 | 2561/2676 | 14345/13841 | KOHBRA/KoGES,MEC,NCC,SeBCS-II,Tianjin |
| rs10889221 | 2918/2324 | 3943/3843 | 5026/5063 | 2563/2673 | 14450/13903 | KOHBRA/KoGES,MEC,NCC,SeBCS-II,Tianjin |
| rs11033111 | 2916/2322 | 1570/1771 | 4990/5021 | 5222/7460 | 14698/16574 | MEC,SeBCS-I,Tianjin |
| rs1156590 | 2743/2165 | 1592/1774 | 4982/5024 | 2552/2672 | 11869/11635 | KOHBRA/KoGES,MEC,NCC,SeBCS-I,SeBCS-II,Tianjin |
| rs12644365 | 2763/2175 | 3939/3840 | 5005/5040 | 2553/2667 | 14260/13722 | KOHBRA/KoGES,MEC,NCC,SeBCS-II,Tianjin |
| rs17273198 | 2888/2282 | 1611/1797 | 4993/5006 | 6460/8997 | 15952/18082 | MEC,SeBCS-I |
| rs2471214 | 2913/2322 | 3954/3852 | 5026/5048 | 3832/4236 | 15725/15458 | KOHBRA/KoGES,MEC,NCC,SeBCS-II |
| rs297709 | 2751/2155 | 3926/3822 | 5028/5055 | 4004/4248 | 15709/15280 | KOHBRA/KoGES,MEC,NCC,SeBCS-II |
| rs3176626 | 2909/2310 | 1610/1797 | 5033/5061 | 2558/2677 | 12110/11845 | KOHBRA/KoGES,MEC,NCC,SeBCS-I,SeBCS-II,Tianjin |
| rs4613895 | 2731/2117 | 1608/1798 | 5035/5062 | 2568/2684 | 11942/11661 | KOHBRA/KoGES,MEC,NCC,SeBCS-I,SeBCS-II,Tianjin |
| rs4732987 | 2772/2178 | 3937/3829 | 5032/5041 | 6437/8987 | 18178/20035 | MEC |
| rs4976412 | 2773/2178 | 3971/3852 | 5086/5041 | 5207/7449 | 17037/18520 | MEC,Tianjin |
| rs5750715 | 2768/2177 | 3944/3849 | 4995/5029 | 2550/2671 | 14257/13726 | KOHBRA/KoGES,MEC,NCC,SeBCS-II,Tianjin |
| rs628583 | 2913/2320 | 3946/3850 | 4999/5021 | 2579/2681 | 14437/13872 | KOHBRA/KoGES,MEC,NCC,SeBCS-II,Tianjin |
| rs6926191 | 2914/2323 | 3948/3852 | 5053/5067 | 2582/2686 | 14497/13928 | KOHBRA/KoGES,MEC,NCC,SeBCS-II,Tianjin |
| rs7107217 | 2916/2319 | 3929/3839 | 4606/4424 | 7348/9831 | 18799/20413 | HongKong |
| rs7443571 | 2918/2324 | 3950/3851 | 5128/5061 | 2648/4777 | 14644/16013 | Guangzhou,MEC,Nanjing,Tianjin |
| rs9383951 | 2916/2319 | 3948/3836 | 5009/5055 | 6117/8296 | 17990/19506 | Tianjin |
| rs9485372 | 2770/2175 | 3930/3818 | 4081/4074 | 5186/7440 | 15967/17507 | HongKong,MEC,Tianjin |
| rs980281 | 2912/2321 | 3966/3844 | 5036/5048 | 3536/5605 | 15450/16818 | Guangzhou,Nanjing,Tianjin |
| ^a^ Number of cases/number of controls | | |  |  |  |  |
