# Supplementary material for: Genome-Wide Association Study in East Asians Identifies Novel Susceptibility Loci for Breast Cancer
Source: PLoS Genet. 2012 Feb 23;8(2):e1002532. doi: 10.1371/journal.pgen.1002532 (PMC3285588; doi:10.1371/journal.pgen.1002532)
Supplement: Text S1 — Supplementary Methods. (DOCX) [file pgen.1002532.s012.docx]

**Supplementary Method**

**Description of Study Participants**

**Shanghai Breast Cancer Study (SBCS):** The SBCS is a population-based case-control study conducted in urban Shanghai, the largest commercial center in China (1;2). For the SBCS-I, subjects were recruited between 1996 and 1998. Through a rapid case-ascertainment system and the population-based Shanghai Cancer Registry, 1,602 eligible breast cancer cases diagnosed during the study period were identified, of which 1,459 cases (91.1%) completed in-person interviews. Cancer diagnoses for all patients were reviewed and confirmed by two senior pathologists. Controls were randomly selected from the general population using the Shanghai Resident Registry, a population registry containing demographic information for all residents of urban Shanghai. The inclusion criteria for controls were identical to those for cases, with the exception of a breast cancer diagnosis. Of the 1,724 eligible controls, 1,556 (90.3%) completed in-person interviews. A structured questionnaire was used to elicit detailed information on demographic factors and known/suspected risk factors for breast cancer. All participants were measured for their current weight, height, and circumference of the waist and hips. All interviews were tape-recorded and reviewed by the field supervisor and quality-control staff to monitor the quality of interview data. Blood samples (10 ml from each woman) were obtained from 1,193 (82%) cases and 1,310 (84%) controls who completed the in-person interview. A sample of exfoliated buccal cells was obtained using cotton swabs from virtually all study participants who did not provide a blood sample. Because DNA yield from buccal cell samples collected in cotton swabs is low, the current study is limited to those who provided a blood sample.

#### Using a protocol similar to the SBCS-I, the SBCS-II recruited 1,989 incident breast cancer cases and 1,989 community controls between 2002 and 2005 with a response rate of 83.7% and 70.4%, respectively. Similar to subject recruitment in the SBCS-I, the majority of newly recruited cases (n=1,932, 97.1%) and controls (n=1,857, 93.4%) provided a blood sample or an exfoliated buccal cell sample to the study. The mouthwash method used in the study was modified from that reported initially by Lum and Le Marchand (3) and provided, on average, approximately 34 μg of DNA per sample. With the exception of age, eligibility criteria for study participation were identical for SBCS-I and SBCS-II. The age range was expanded from 25 to 65 years in SBCS-I, to 25 to 70 years in SBCS-II.

**Shanghai Breast Cancer Survival Study (SBCSS)** **and** **Shanghai Endometrial Cancer Study (SECS):** The SBCSS also used the population-based Shanghai Cancer Registry to identify newly-diagnosed breast cancer cases for the study (2). A total of 6,303 cases were diagnosed between April 1, 2002, and December 31, 2006, and were approached for the study approximately six months after cancer diagnosis; 5,046 were recruited (response rate: 80.1%). In-person interviews were conducted to collect information on known breast cancer risk factors and anthropometrics by using a protocol and questionnaire similar to that used in the SBCS. Buccal cell samples were collected from 96% of study participants using the modified mouthwash method described above. Because of a time overlap in subject recruitment in the SBCS-II and the SBCSS, 1,469 breast cancer patients participated in both studies. The remaining 3,466 SBCSS cases were included in the current study.

Controls for this group of cases were derived from the SECS (2), a population-based case-control study conducted between 1997 and 2003, a time period that overlapped with the SBCS and SBCSS. With the exception of a few questions related specifically to breast or endometrial cancer risk, the questionnaires used in the SECS and the SBCS were virtually identical. Using a protocol similar to the one used in the SBCS, eligible cases were identified through the population-based Shanghai Cancer Registry and controls were randomly selected from the general population of Shanghai using the Shanghai Resident Registry and were age frequency-matched to cases. Women with a history of cancer or hysterectomy were not eligible. In-person interviews were conducted by trained interviewers to collect detailed information on demographic factors as well as known and suspected risk factors. Of the study participants who completed an in-person interview, 1,039 controls provided a blood sample or buccal cell sample using the mouthwash method, and these women were included in the current study.

**Shanghai Women’s Health Study (SWHS):** The SWHS is a population-based prospective cohort study of approximately 75,000 adult women who were recruited between 1997 and 2000 (4). At the baseline recruitment, a roster of all women aged 40 to 70 years was obtained from the resident registry offices in the study communities. Of the 81,170 eligible women, 75,221 participated in the study, with a participation rate of 92.7%. Detailed exposure data were collected during the baseline survey through an in-person interview. Body weight/height and circumferences of the waist and hips were measured. Among those who completed the survey, 56,831 (75.8%) donated a blood sample, and 65,754 (87.7%) donated a urine sample. An exfoliated buccal cell sample was collected from an additional 8,934 (49.3%) of the 18,111 subjects who did not provide a blood sample at baseline. Therefore, we have genomic DNA from about 88% of cohort members.

The cohort has been followed by a combination of record linkage and active follow-ups. Every two years, an interviewer visits the last known address of each living cohort member and records details of the interim health history, including cancer and several other chronic diseases that occurred since the last in-person contact. In addition to the interim health history, the survey questionnaire also includes a module to obtain information related to selected lifestyle factors, including a FFQ. Data routinely collected by the cancer registry and death certificates are also used to assure a timely and complete ascertainment of new cancer cases and deceased subjects in the study cohort. All possible matches are checked manually and verified through home visits. For cohort members who are diagnosed with cancer, information on date and hospital of diagnosis is collected. Copies of medical charts from the diagnostic hospital are obtained to verify the diagnosis and collect detailed information on the pathology characteristics of the tumor. In addition, pathology slides and tumor tissue blocks are being collected to verify cancer diagnosis and for future studies of biomarkers. The first follow-up survey was conducted from 2000 to 2002. Approximately 99.8% of cohort members (or their next of kin, if subjects were deceased) were interviewed. The response rates were 98.7% for the second follow-up survey (2002-04), 96.7% for the third follow-up survey (2004-07), and approximately 93% for the fourth follow-up (2007-2010). For non-respondents, cancer diagnosis and vital status can still be identified through the linkage of data from cancer and vital statistics registries, and thus ascertainment for cancer outcomes and total mortality is virtually complete in this cohort. Breast cancer patients identified in the SWHS and non-cases were included in the current study.

**Tianjin Study** (5): This hospital-based case-control study included 1,532 breast cancer patients and 1,583 healthy female controls. Patients were histopathologically diagnosed with primary breast cancer and were consecutively recruited from the Department of Breast Surgery of Tianjin Cancer Institute and Hospital, China, between December 2004 and December 2008. Age (±5 years) frequency-matched controls were recruited from women who attended health screenings at the Center of Health Examination in the same hospital during the same period and had no evidence of cancer. Controls with diseases of the cardiovascular, respiratory, digestive, urinary, reproductive, or endocrine systems were also excluded. All breast cancer patients and controls were genetically unrelated, ethnic Han Chinese women who were permanent residents of the urban area of Tianjin. ER and PR status was determined using immunohistochemical analysis by breast cancer pathologists in the Department of Pathology. After giving informed consent, each participant was interviewed face-to-face by trained interviewers using a pre-tested questionnaire to obtain information on demographic data, menstrual and reproductive history, lifestyle, environmental exposures, and family history of cancer. After the interview, a 5-ml venous blood sample was collected from each participant and used for DNA extraction and genotyping. The study protocol was approved by the Tianjin Cancer Institute and Hospital review committee.

**Nanjing Study (6)**: This hospital-based case-control study included 1,446 breast cancer cases and 1,439 cancer-free controls. Incident breast cancer patients were consecutively recruited from the First Affiliated Hospital of Nanjing Medical University, the Cancer Hospital of Jiangsu Province, and the Gulou Hospital, Nanjing, China, between January 2004 and July 2008. Exclusion criteria included self-reported prior history of cancer, metastasized cancer from other organs, and previous radiotherapy or chemotherapy. All included breast cancer cases were newly diagnosed and histopathologically confirmed without restrictions of age. Cancer-free controls, frequency-matched to the cases on age (±5 years) and residential area (urban or rural), were randomly selected from a cohort of >30,000 participants in a community-based screening program for non-infectious diseases conducted from 2004 to 2006 in Jiangsu Province, China. All participants were genetically unrelated, ethnic Han Chinese women. After providing informed consent, each woman was interviewed face-to-face by trained interviewers using a standard questionnaire to obtain information on demographic characteristics, menstrual and reproductive history, environmental exposure history, and family history of any cancer in first-degree relatives (parents, siblings, and children). Data collection methods were similar for cases and controls. After the interview, each subject provided 5 ml of venous blood. The ER and PR status of breast cancers was determined from the results of immunohistochemical examinations recorded in the medical records of the hospitals. This study was approved by the Institutional Review Board of Nanjing Medical University.

**Taiwan Study (7):** This case-control study is part of an on-going, cooperative study aimed at understanding the causes of breast cancer in Taiwan, which is characterized by low incidence, early tumor onset, hormone dependency, and novel genomic alterations. The study included 1,001 female breast cancer patients and 1,013 healthy female controls. All breast cancer patients had pathologically confirmed incident primary breast cancer and were diagnosed and treated at the Tri-Service General Hospital or the Changhua Christian Hospital between March 2002 and August 2005. The participation rate was over 90%. Patients with inadequate blood specimens were excluded from the study. Women included in the study were similar to those excluded in the distribution of major breast cancer risk factors. Because these are two of the major breast cancer clinics in northern and central Taiwan, patients recruited for the study accounted for a significant proportion (~40%) of all breast cancer cases diagnosed during the study period in these regions. Controls were randomly selected from women attending the health examination clinics of the same hospitals during the same period. These women underwent a one-day comprehensive health examination (including regular breast screening using X-ray mammography and ultrasonic examination), and those showing any evidence of breast cancer, suspicious precancerous lesions of the breast, or other cancers were excluded from the control group. Almost all women (>95%) initially identified as potential controls participated in the study, and the controls accounted for ~20% of all women attending the clinics. No significant differences in socioeconomic status were found between those included and those excluded from the study. Informed consent was obtained from all study participants before collection of epidemiologic data through in-person interviews. At the completion of each interview, blood was taken for DNA isolation and genotyping. Two experienced research nurses were assigned to administer a structured questionnaire to cases and controls. The information collected included age, family history of breast cancer, age at menarche and/or menopause, history of full-term pregnancy, menopausal status, and body mass index.

**Hong Kong Study (8)**: This is a hospital-based study consisting of women with incident breast cancer, recruited during the period of June 2003 to March 2009 from patients attending follow-up surgical and oncology outpatient clinics at three major public hospitals on Hong Kong Island (Queen Mary Hospital), and Kowloon (Queen Elisabeth Hospital and Kwong Wah Hospital). All participants completed face-to-face interviews. Control participants matched for age on 10-year intervals were recruited from outpatients attending the general gynecological clinic at Queen Mary Hospital and from the Well-Women Clinic at Kwong Wah Hospital, who had no personal history of cancer. They were also questioned about any family history of breast and/or ovarian cancer. About 70% of cases and controls interviewed agreed to participate in this project. Blood samples were obtained from 517 cases and 651 controls, which were subsequently used for DNA extraction by proteinase K digestion followed by conventional phenol-chloroform-ethanol extraction. The protocol was approved by the Institutional Review Boards of the University of Hong Kong Hospital Authority. Patient consent was obtained for study participation and blood collection.

**Guangzhou Study:** This ongoing case-control study is aimed at understanding the causes of breast cancer in Guangzhou area of southern China. The Patients were histopathologically diagnosed with primary breast cancer and were consecutively recruited from the Affiliated Cancer Center, the First Affiliated Hospital and the Second Affiliated Hospital of Sun Yat-sen University, Guangzhou, China, between October 2008 and June 2010. A total of 915 eligible breast cancer cases during the study period completed in-person interviews with response rates of 75% to 95% depending on the hospitals. The ER and PR status was determined with immunohistochemistry by pathologists in the corresponding hospitals. Women with metastasized breast cancer or previous history of other cancers were excluded. Cancer-free controls, frequency-matched to the cases on age (±5 years) were identified from the primary care databases of the same hospitals during the same period. Women who self-reported a history of cancer were excluded. Of the eligible controls, 939 (78.3%) completed in-person interviews. All subjects must have resided in Guangzhou area for at least 5 years and they were genetically unrelated, ethnic Han Chinese women. A structured questionnaire was used to obtain information on demographic data, menstrual and reproductive history, environmental exposure history, and family history of any cancer in first-degree relatives. Blood samples (5 ml from each women) were obtained from 838 (91.6%) cases and 865 (92.1%) controls who completed the in-person interview. Informed consent was obtained for the interview and speciman collection. The Ethical Committee of the School of Public Health at Sun Yat-sen University approved this study.

**The Multiethnic Cohort Study** **(MEC) (9)**: The MEC is a population-based prospective cohort study that was initiated between 1993 and 1996 and includes subjects from various ethnic groups – African-Americans and Latinos primarily from California (mainly Los Angeles) and Native Hawaiians, Japanese-Americans, and European-Americans primarily from Hawaii. State driver's license files were the primary sources used to identify study subjects in Hawaii and California. Additionally, in Hawaii, state voter's registration files were used, and in California, Health Care Financing Administration (HCFA) files were used to identify additional African-American men. All participants (n = 215,251) returned a 26-page, self-administered baseline questionnaire that obtained general demographic, medical, and risk-factor information such as ethnicity, prior medical conditions, family history of various cancers, dietary exposures, smoking, physical activity, body mass index (BMI), and for women, reproductive history and exogenous hormone use. All participants were 45 to 75 years of age at baseline. In the cohort, incident cancer cases are identified annually through cohort linkage to population-based cancer Surveillance, Epidemiology, and End Results (SEER) registries in Hawaii and Los Angeles County as well as to the California Cancer Registry. Information on stage of disease and estrogen and progesterone receptor status was also obtained through the SEER registries. Blood-sample collection in the MEC began in 1994, targeting incident breast cancer cases and a random sample of study participants to serve as controls for genetic analyses. In the present study, incident cases were defined as those diagnosed with invasive breast cancer after enrollment through December 31, 2005, in Hawaii, and January 31, 2006, in California. Cases were older than 45 years of age and consisted primarily of postmenopausal women. Women with a previous diagnosis of breast cancer identified by SEER or self-reported at baseline were excluded. Controls were women without a breast cancer diagnosis through December 31, 2005, in Hawaii, and January 31, 2006, in California. Controls were frequency-matched to cases on ethnicity and the case's age at diagnosis in five-year intervals. The nested breast cancer case-control study consists of 889 invasive Japanese-American breast cancer cases and 830 controls, and has been utilized previously for numerous candidate gene association studies in the MEC. This study was approved by the Institutional Review Boards at the University of Southern California and the University of Hawaii. Informed consent was obtained from all study participants.

#### Nagoya Study (Hospital-based Epidemiologic Research Program at Aichi Cancer Center, HERPACC-II) (10): This is a hospital-based, comprehensive epidemiologic research program at the Aichi Cancer Center (ACC), Japan. All first-visit outpatients 20-79 years of age at the ACC from December 2000 to November 2005 were asked to participate in the HERPACC-II. A total of 29,736 eligible patients were approached, and 28,766 participated in the study, with a response rate of 96.7%. Subjects completed a self-administered questionnaire about their lifestyle and demographic characteristics and to provide blood samples. Dietary habits were investigated using a 47-item semi-quantitative food frequency questionnaire. ER status for cases was taken from medical records. ER status is routinely determined by pathologists by using commercially based immunohistochemistry tests at the ACC. Case status was confirmed by linkage of the HERPACC-II database and the hospital-based cancer registry database. 1,850 histologically-confirmed breast cancer cases were identified, and 644 were selected for the Asia Breast Cancer Consortium analysis based on availability of DNA samples. Of 14,260 non-cancer subjects in the HERPACC-II database, 644 subjects matched for age and menopausal status were randomly selected. The study protocol was approved by the Institutional Review Board at the ACC (Nagoya, Japan).

#### Nagano Breast Cancer Study (11): This multicenter, hospital-based case-control study was conducted from May 2001 to September 2005 at four hospitals in Nagano Prefecture, Japan. The cases – a consecutive series of women ages 20-74 years with newly diagnosed, histologically confirmed invasive breast cancer – were admitted to the four hospitals during the survey period. Of the 412 eligible patients, 405 (98%) agreed to participate. Healthy controls were selected from medical checkup examinees in two of the hospitals who were confirmed as not having any cancer, with one control matched for each case by age (within three years) and residential area during the study period. Among potential control subjects, one declined to participate. Written informed consent was obtained from 405 matched pairs. Because two controls refused to provide blood samples, the analysis was restricted to 403 matched pairs. Participants completed a self-administered questionnaire, which included questions on demographic characteristics, anthropometric factors, smoking habits, family history of cancer, physical activity, medical history, and menstrual and reproductive history. Dietary habits were investigated using a 136-item semi-quantitative food-frequency questionnaire (FFQ) , which was developed and validated in the Japanese population. The ER and PR status of the patient’s breast cancer tissue was obtained from medical records. Hormone receptor positivity values were determined either as specified by the laboratory that performed the assay, in accordance with the laboratory’s written interpretation thereof, or both. The study protocol was approved by the Institutional Review Board of the National Cancer Center (Tokyo, Japan).

**Seoul Breast Cancer Study (SeBCS) (12;13)(Lee, 2005 #1128)**: The cases consisted of a consecutive series of incident breast cancer patients admitted to two teaching hospitals located in Seoul, Korea. The control subjects consisted of non-cancer patients admitted to the same hospitals as the cases in the same period and of the healthy women who participated in the community health examination provided by the National Health Insurance Corporation (NHIC). Newly diagnosed with histologically confirmed breast cancer cases (n = 4,109) and controls (n=4,980) were recruited in the Seoul National University Hospital and Asan medical center between 1995 and 2006. After obtaining written informed consent, blood was drawn into 10-mL heparinized tubes and stored at -70 °C until genotyping. A questionnaire was given by trained interviewers to collect the information on demographic characteristics, education, marital status, family history of breast cancer, reproductive factors, menstruation, and lifestyles including alcohol consumption, smoking, and diet. A total of 3,136 breast cancer cases (2,359 subjects for Stage II and 777 subjects for Stage IV) and 1,104 controls (Stage IV) with sufficient amount of DNA were selected and analyzed. For cases, a retrospective chart review was used to collect clinical information including tumor features and disease severity including tumor size, lymph node invasion, distant organ metastasis, hormone receptor status, and hormone receptor therapy. The main disease of control subjects included infection, stone of gall bladder/bile duct, acute appendicitis, hemorrhoid, benign breast cancer and the others. Controls from the health examination program were recruited between 2006 and 2007. Standardized questionnaire were administered by trained interviewers, including socio-demographics characteristics, past medical history, medication usage, family history, lifetime consumption of alcohol and tobacco, diet, physical activity, and reproductive factors. Various biological specimens were collected in the biorepository according to the standardized protocol. Of 32,887 subjects, 2,052 women from the same catchment area as the cases and matched on age (5-year increments) were selected and analyzed.

**Korea Genome Epidemiology Study (KoGES) (14)**: The KoGES is ongoing study since 2001 to investigate major genetic and environmental factors for common diseases in the Korean population. Of 10,038 subjects surveyed at baseline enrollment in 2001, 1,536 women with sufficient DNA concentrations were analyzed. Of 7,861 subjects recruited from 2005 to 2006, 1,673 women were analyzed. Thus, a total of 3,209 control subjects were selected and analyzed for Stage IV.

**Korean Hereditary Breast Cancer (KOHBRA) (15)**: The KOHBRA study is an ongoing cohort study since 2007 to examine high risk groups for hereditary breast cancer such as female breast cancer patients with a family history, ovarian cancer, or other coincidental cancers, male breast cancer patients, and family members of breast cancer patients with *BRCA*1/2 mutation4. We finally selected 1,397 female cancer patients without *BRCA*1, 2 mutation among KOHBRA subjects recruited in 2007-2009.

**Korea NCC study:** Newly diagnosed breast cancer patients (cases, n=505) were recruited from the Breast Cancer Clinic at the Asan Medical Center in Seoul, Korea between February 2006 and July 2010. Each case member had received a histologically confirmed diagnosis of their first primary breast cancer and participated in the study before the treatment was started. Ineligibility criteria were a previous malignancy (at either the same site or a different site) and an age greater than 80 years. The hospital controls (n=505) were women free of any malignant neoplasms and free of any clinical, biochemical, or hematological manifestations of cardiovascular, hepatic, renal, or endocrinal disorders. All case and control subjects completed a questionnaire on lifestyle and dietary intake and provided blood samples. Informed consent was obtained from all subjects after a full explanation of the study, which had been previously approved by the institutional review board of the Korea National Cancer Center. Both case and control subjects were interviewed by one trained interviewer who was unaware of the subject’s status. Using both a non-dietary questionnaire and a 95-item semi-quantitative food frequency questionnaire, information was collected on socio-demographic characteristics, anthropometric measures, individual medical history, family cancer history, and dietary factors detailing their usual food intake over the year prior to enrolment in the study. Socio-demographic characteristics included education level, occupation, cigarette smoking status, alcohol consumption, and physical activity. Pathological and laboratory data for each subject were collected, recorded, and entered into an epidemiological database. Medical charts and pathology reports were examined to ensure that control subjects had no known history of cancer. A peripheral venous blood sample (20 ml aliquot in an anticoagulant tube) was obtained from each enrolled subject. Laboratory assays of the blood samples were performed before the initiation of any treatment or therapy. Blood samples were wrapped in aluminum foil to protect against photo-oxidation, and transported to the laboratory without revealing the subject’s case/control status prior to performing the antioxidant micronutrient assay. After separating plasma, samples were stored at -80°C until assayed.

Reference List

1. Gao YT, Shu XO, Dai Q, Potter JD, Brinton LA, Wen W, Sellers TA, Kushi LH, Ruan Z, Bostick RM, et al. Association of menstrual and reproductive factors with breast cancer risk: results from the Shanghai Breast Cancer Study. Int.J.Cancer 2000 Jul 15;87(2):295-300

2. Zheng W, Long J, Gao YT, Li C, Zheng Y, Xiang YB, Wen W, Levy S, Deming SL, Haines JL, et al. Genome-wide association study identifies a new breast cancer susceptibility locus at 6q25.1. Nat.Genet. 2009 Mar;41(3):324-8. PMCID:PMC2754845

3. Lum A, Le ML. A simple mouthwash method for obtaining genomic DNA in molecular epidemiological studies. Cancer Epidemiol.Biomarkers Prev. 1998 Aug;7(8):719-24

4. Zheng W, Chow WH, Yang G, Jin F, Rothman N, Blair A, Li HL, Wen W, Ji BT, Li Q, et al. The Shanghai Women's Health Study: rationale, study design, and baseline characteristics. Am.J.Epidemiol. 2005 Dec 1;162(11):1123-31

5. Zhang L, Gu L, Qian B, Hao X, Zhang W, Wei Q, Chen K. Association of genetic polymorphisms of ER-alpha and the estradiol-synthesizing enzyme genes CYP17 and CYP19 with breast cancer risk in Chinese women. Breast Cancer Res.Treat. 2009 Mar;114(2):327-38

6. Liang J, Chen P, Hu Z, Zhou X, Chen L, Li M, Wang Y, Tang J, Wang H, Shen H. Genetic variants in fibroblast growth factor receptor 2 (FGFR2) contribute to susceptibility of breast cancer in Chinese women. Carcinogenesis 2008 Dec;29(12):2341-6

7. Ding SL, Yu JC, Chen ST, Hsu GC, Kuo SJ, Lin YH, Wu PE, Shen CY. Genetic variants of BLM interact with RAD51 to increase breast cancer susceptibility. Carcinogenesis 2009 Jan;30(1):43-9

8. Chan KY, Liu W, Long JR, Yip SP, Chan SY, Shu XO, Chua DT, Cheung AN, Ching JC, Cai H, et al. Functional polymorphisms in the BRCA1 promoter influence transcription and are associated with decreased risk for breast cancer in Chinese women. J.Med.Genet. 2009 Jan;46(1):32-9. PMCID:PMC2782922

9. Haiman CA, Garcia RR, Hsu C, Xia L, Ha H, Sheng X, Le ML, Kolonel LN, Henderson BE, Stallcup MR, et al. Screening and association testing of common coding variation in steroid hormone receptor co-activator and co-repressor genes in relation to breast cancer risk: the Multiethnic Cohort. BMC.Cancer. 2009 Jan 30;9:43.:43

10. Hamajima N, Matsuo K, Saito T, Hirose K, Inoue M, Takezaki T, Kuroishi T, Tajima K. Gene-environment Interactions and Polymorphism Studies of Cancer Risk in the Hospital-based Epidemiologic Research Program at Aichi Cancer Center II (HERPACC-II). Asian Pac.J.Cancer Prev. 2001;2(2):99-107

11. Itoh H, Iwasaki M, Hanaoka T, Kasuga Y, Yokoyama S, Onuma H, Nishimura H, Kusama R, Tsugane S. Serum organochlorines and breast cancer risk in Japanese women: a case-control study. Cancer Causes Control 2009 Jul;20(5):567-80

12. Choi JY, Lee KM, Park SK, Noh DY, Ahn SH, Yoo KY, Kang D. Association of paternal age at birth and the risk of breast cancer in offspring: a case control study. BMC.Cancer. 2005 Oct 31;5:143.:143

13. Lee KM, Choi JY, Park SK, Chung HW, Ahn B, Yoo KY, Han W, Noh DY, Ahn SH, Kim H, et al. Genetic polymorphisms of ataxia telangiectasia mutated and breast cancer risk. Cancer Epidemiol.Biomarkers Prev. 2005 Apr;14(4):821-5

14. Cho YS, Go MJ, Kim YJ, Heo JY, Oh JH, Ban HJ, Yoon D, Lee MH, Kim DJ, Park M, et al. A large-scale genome-wide association study of Asian populations uncovers genetic factors influencing eight quantitative traits. Nat.Genet. 2009 May;41(5):527-34

15. Han SA, Park SK, Hyun AS, Hyuk LM, Noh DY, Kim LS, Noh WC, Jung Y, Sang KK, Kim SW, et al. The Korean Hereditary Breast Cancer (KOHBRA) Study: Protocols and Interim Report. Clin.Oncol.(R.Coll.Radiol.). 2011 Apr 14;
